# Prosocial Influence and Opportunistic Conformity in Adolescents and Young Adults

**DOI:** 10.1177/0956797620957625

**Published:** 2020-11-23

**Authors:** Gabriele Chierchia, Blanca Piera Pi-Sunyer, Sarah-Jayne Blakemore

**Affiliations:** 1 Division of Psychology and Language Sciences, Institute of Cognitive Neuroscience, University College London; 2 Department of Psychology, University of Cambridge

**Keywords:** social influences, conformity, cooperation, adolescent development, prosocial decisions, open data, open materials

## Abstract

Adolescence is associated with heightened social influence, especially from peers. This can lead to detrimental decision-making in domains such as risky behavior but may also raise opportunities for prosocial behavior. We used an incentivized charitable-donations task to investigate how people revise decisions after learning about the donations of others and how this is affected by age (*N* = 220; age range = 11–35 years). Our results showed that the probability of social influence decreased with age within this age range. In addition, whereas previous research has suggested that adults are more likely to conform to the behavior of selfish others than to the behavior of prosocial others, here we observed no evidence of such an asymmetry in midadolescents. We discuss possible interpretations of these findings in relation to the social context of the task, the perceived value of money, and social decision-making across development.

Adolescence, defined as the period of life between puberty and adulthood, is associated with heightened social influence, especially from peers (Blakemore, 2018). Existing studies on peer influence in adolescence have tended to focus on social influence on risky decision-making and risk perception (Reniers et al., 2017) and have more recently been extended to other processes, including cognitive performance (Wolf, Bazargani, Kilford, Dumontheil, & Blakemore, 2015) and hypothetical prosocial behavior (Foulkes, Leung, Fuhrmann, Knoll, & Blakemore, 2018).

However, it remains unclear how such age effects on prosocial influence may apply to situations in which participants incur real costs to help other people. This is important for a number of reasons. First, hypothetical and real prosocial behaviors have been shown to be frequently unrelated (e.g., Böckler, Tusche, & Singer, 2016). Second, real costs are known to introduce an asymmetry in the way adults conform to the behavior of other individuals, leading them to preferentially adapt to selfish norms (which involve monetary benefits), relative to prosocial ones (which involve monetary costs; Charness, Naef, & Sontuoso, 2019; Croson & Shang, 2008; Dimant, 2019). On the other hand, relative to adults, adolescents have been frequently suggested to be more concerned with conforming and fitting in with other people, that is, to be more wary of the social costs and benefits of their actions (Blakemore, 2018), possibly at the expense of monetary concerns. This might result in adolescents conforming to prosocial and selfish norms in a different way than adults. In this study, we focused on prosocial decisions that are costly to oneself but beneficial to others. In particular, we investigated how prosocial influence, the tendency to engage in prosocial behavior after observing it in other people, is modulated by age and whether this further depends on the source of influence (i.e., whether one is influenced by peers, nonpeers, or a computer) and on the direction of influence (i.e., whether others are more or less prosocial than oneself).

So far, the relationship between social influence and adolescent decision-making has largely been investigated in the context of risk taking (Blakemore, 2018; Knoll, Magis-Weinberg, Speekenbrink, & Blakemore, 2015; Reiter, Suzuki, O’Doherty, Li, & Eppinger, 2019; Reniers et al., 2017). This is a natural starting point, given that excessive risk taking, such as binge drinking, smoking, substance use, and reckless driving, is a prevalent source of vulnerability during adolescence (Steinberg, 2008). However, social influence is not restricted to risky decisions (Van Hoorn, Van Dijk, Güroğlu, & Crone, 2016). It is also proposed to play a fundamental role in spreading and maintaining prosocial norms (Kraft-Todd, Yoeli, Bhanot, & Rand, 2015). Indeed, prosocial influence has been observed in a variety of domains, including contributions to public goods and charitable giving, helping, and fairness in economic games (Croson & Shang, 2008; Kraft-Todd et al., 2015; Nook, Ong, Morelli, Mitchell, & Zaki, 2016; Wei, Zhao, & Zheng, 2016).

Prosocial influence is observed in early childhood in humans (Schmidt & Tomasello, 2012) as well as in nonhuman primates (Berthier & Semple, 2018). Adolescents are no exception (Choukas-Bradley, Giletta, Cohen, & Prinstein, 2015; van Goethem, van Hoof, van Aken, Orobio de Castro, & Raaijmakers, 2014; Van Hoorn et al., 2016). For example, the mere presence of peers increased monetary contributions by adolescents in a public-goods game (Van Hoorn et al., 2016). Similarly, volunteering by adolescents has been found to be influenced by whether their peers also volunteer (Choukas-Bradley et al., 2015; van Goethem et al., 2014). It follows that prosocial influence is prevalent across ages, but less is known about whether, as in the domain of risk, prosocial influence is heightened during adolescence. One recent study showed that prosocial influence decreased linearly with age between early adolescence and adulthood (Foulkes et al., 2018). However, this study focused on hypothetical prosocial decisions, which can be unrelated to incentivized decisions (e.g., Böckler et al., 2016). It thus remains unclear whether age might similarly affect prosocial influence in decision-making that involves real monetary incentives.

In fact, these monetary incentives plausibly underlie a known opportunistic asymmetry in the way adults adapt their prosocial behavior to the prosocial behavior of others (Charness et al., 2019), leading them to conform to the behavior of others more when this aligns with their own material self-interest and, thus, to preferentially decrease rather than increase their prosocial behavior. For example, adults adjusted their contributions to a public good (i.e., a public radio station) more in line with other individuals’ contributions when informed that others had contributed less than them, compared with when others had given more (Croson & Shang, 2008). Similarly, adults have been shown to conform more to antisocial relative to prosocial behavior (Dimant, 2019) and to align their trust-related decisions with others more when this allows them to earn more (Charness et al., 2019).

Finally, none of the studies above controlled for nonsocial-influence effects, which are known to have an impact on decision-making in adults. For example, adults adapted their decisions to those of a computer when making incentivized decisions (Moutoussis, Dolan, & Dayan, 2016), even when they were informed that the agent they were observing was simulated. This introduces the possibility that some of the previously reported social-influence effects might have been conflated with nonsocial-influence effects, such as more automatic or narrow forms of imitation (Nook et al., 2016), priming effects (Moutoussis et al., 2016), and anchoring effects (e.g., Wilson, Houston, Etling, & Brekke, 1996).

We aimed to fill these gaps by addressing the following hypotheses. First, we aimed to extend the *age-dependent-influence hypothesis*—previously observed in the domains of risk (Knoll, Leung, Foulkes, & Blakemore, 2017; Knoll et al., 2015) and hypothetical prosocial behavior (Foulkes et al., 2018)—to situations in which prosocial behavior has real monetary costs. Second, we tested the *peer-influence hypothesis* by introducing a teenager-versus-adults distinction in the source of influence, as in previous studies (Foulkes et al., 2018; Knoll et al., 2017; Knoll et al., 2015; Reiter et al., 2019; van Goethem et al., 2014). Third, we tested a *social-influence hypothesis* by comparing social influence from other people (teenagers or adults) with nonsocial influence from a computer (Moutoussis et al., 2016). Finally, we investigated a *direction-of-influence hypothesis* by assessing how participants responded when they learned that other people donated more or less than them (Knoll et al., 2017; Reiter et al., 2019).

## Method

### Participants

Previous research suggests that developmental effects on decision-making during adolescence range between small and medium (Defoe, Dubas, Figner, & van Aken, 2015; Knoll et al., 2015; Reiter et al., 2019). In particular, previous studies employing a similar paradigm to the one used here (Knoll et al., 2015; Reiter et al., 2019) suggest that recruiting between 100 and 250 participants between early adolescence and early adulthood should suffice to detect these effects. We recruited 220 participants (106 female) between the ages of 11 and 35 years (see Table 1 for participant demographics; see also Section 1 in the Supplemental Material available online). Participants were divided into three age groups for comparability with previous research (Foulkes et al., 2018; Knoll et al., 2017; Knoll et al., 2015; Reiter et al., 2019): young adolescents (11–14 years), midadolescents (15–18 years), and adults (23–35 years). All analyses were additionally conducted using age as a continuous variable, to avoid any grouping criteria. Participants younger than 18 years were recruited through schoolwide announcements from teachers within participating schools. Group sessions took place in school computer rooms, and group size varied between 1 and 14 pupils per classroom. Each participant completed the task on an individual computer, and desks were sufficiently spaced apart so that participants would be unable to read the screens of the other participants. Participants older than 18 years were recruited through University College London’s subject-pool recruitment system. Sessions took place in groups at the university’s computer cubicles, and group size varied between 1 and 4. Adult participants and parents of participants younger than 18 years provided informed consent. All procedures were approved by University College London’s ethics committee (Approval Code 3453/001). The study was not preregistered. Deidentified data, stimuli, and scripts are available on OSF at https://osf.io/3e9s6/.

**Table 1.** Participant Demographics

Variable	Young adolescents (*n* = 65)	Midadolescents (*n* = 86)	Adults (*n* = 69)
Age (years)			
Range	11–14	15–18	23–35
*M*	12.91	16.60	26.45
*SD*	0.84	0.94	3.43
Gender (*n*)			
Female	34	39	33
Male	31	47	36

### Prosocial-influence task

The study employed a 3 (age: young adolescent, midadolescent, adult; between subjects) × 3 (source: adults, teenagers, computer; within subjects) × 2 (direction: prosocial influence, selfish influence; within subjects) repeated measures design.

To measure prosocial behavior, we adapted a charitable-donation task (e.g., Böckler et al., 2016) in which participants were allotted 50 tokens and asked to decide how many, if any, they wished to donate to a number of charities. Participants were informed that tokens had real monetary value, and consequently, prosocial behavior was costly. Specifically, we informed participants that one random charity would be selected at the end of the session and that any tokens not donated to that charity would be converted to money and paid to them. This occurred as stated. As in previous studies, participants were informed that tokens were worth money, but they were not informed about the exchange rate (e.g., Geier, Terwilliger, Teslovich, Velanova, & Luna, 2010). We did this to avoid selecting an exchange rate that may have been relevant to only a subsample of participants and to reduce the possibility that participants would mentally convert tokens into money, thus freeing working memory for the task. In addition, at the end of the experiment, we asked participants to provide a rating indicating how much money they thought a single token was worth. We observed no age differences in the guesses of such exchange rates (for details, see Section 2 in the Supplemental Material).

**Fig. 1.**
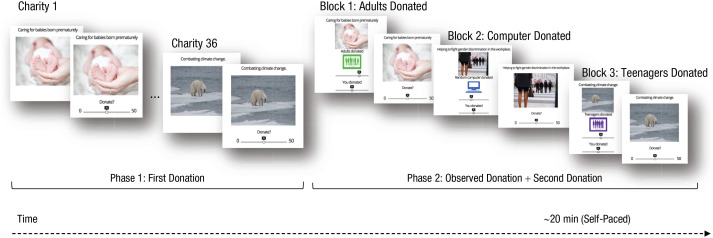
Prosocial-influence task. In Phase 1, participants decided how many tokens, if any, to donate to each of 36 charities. For each charity, they were given 50 tokens to allocate to that charity as they wished, knowing that their donation to one of the charities would be randomly selected at the end of the study, converted into money, and paid. Prosocial behavior was thus costly. In Phase 2, participants observed the average donation made by other donors (teenagers, adults, or a computer) to the same charities and were simultaneously reminded how much they had previously donated themselves. They were then requested to donate to the charity a second time.

To investigate prosocial influence, we divided each of 36 donation trials into two phases (Fig. 1). In Phase 1, participants decided how many tokens to donate to each of 36 different charities, without knowing anything about how much other donors had given (we henceforth refer to these as *first donations*). In Phase 2, participants first observed how much other donors had given to the same charities and were then requested to donate again. There was no time limit to any decisions. Our main variable of interest was whether participants changed their donations from Phase 1 to Phase 2, adjusting them to the donations they observed. In particular, we investigated how the likelihood of prosocial influence was modulated by participants’ age, by the source of influence, and by the direction of influence.

The observed donations could come from one of three supposed sources: the average donation of a group of adults, the average donation of a group of teenagers who had previously taken part in the study, or a randomly generated donation by a computer. These three source-related levels were included in blocks and presented in a counterbalanced order between participants.

As for the direction of influence, the specific donations that participants observed were generated according to an adaptive algorithm that was designed to balance the number of prosocial-influence trials, in which other donors had given more generously than the participant, and selfish-influence trials, in which other donors had given more selfishly than the participant. These conditions were included to potentially induce more prosocial or selfish behavior, respectively (Nook et al., 2016; Wei et al., 2016), and thus to assess possible age effects on opportunistic conformity (Charness et al., 2019; Croson & Shang, 2008; Dimant, 2019). Specifically, the observed donation was a random number in the relevant interval: observed donation ∈ [(donation 1 + 1), 45] for prosocial-influence trials and observed donation ∈ [(donation 1 – 1), 5] for selfish-influence trials. These intervals were capped at 5 and 45, respectively, to avoid implausible observed donations. These capping rules were, however, relaxed when participants displayed skewed donations in Phase 1 (e.g., most donations at ceiling or floor in Phase 1; see Section 3 in the Supplemental Material).

#### Stimuli

In both phases of the prosocial-influence task, participants indicated their donation by moving a slider on a bar with “0” and “50” written at the two extremes. A cursor on top of the bar indicated the current number of tokens, allowing participants to be precise if they wished to. The initial position of the cursor was always set to 25 to provide an unbiased anchor. First and second donations were probed in different phases to obtain an entirely unbiased estimate of participants’ baseline donation behavior (i.e., to avoid any possible cross-item influence). In Phase 2, in addition to a bar indicating the observed donation of other donors, a second bar reminded participants of their own previous donation. This was done to avoid any confounding effects related to forgetting one’s initial donation (Fig. 1).

For each participant, the 36 charities were randomly selected from a set of 120 possible charities dedicated to social, health, or environmental missions (see Section 4 in the Supplemental Material). This variety of charities was adopted to decorrelate prosocial-influence effects from any particular charity contents. Each charity was represented by an image and a brief sentence (*M* = 47 characters, *SD* = 11) related to the charity’s mission. The images were drawn from a number of online image platforms (e.g., Google images) and were all labeled “free for reuse.” All stimuli are available on OSF at https://osf.io/3e9s6/. Stimuli never referred to charity names or logos, to reduce any political connotations or legal implications. The task was implemented on Gorilla (https://gorilla.sc/; Anwyl-Irvine, Massonnié, Flitton, Kirkham, & Evershed, 2020). It can be sampled at https://gorilla.sc/openmaterials/133819 and can be freely cloned.

#### Suspicion

After the prosocial-influence task, participants were probed for any suspicion of deception with a single open-ended question: “Did you feel that any aspect of the task was strange in any way? If so, can you briefly describe what seemed strange? If not, simply respond No.” Nine participants (one young adolescent, two midadolescents, six adults) expressed potential doubts about the veracity of donating to charity or the fact that the observed donations really came from the stated sources. Results were qualitatively unaffected by the exclusion of these participants.

### Abstract-reasoning task

To investigate effects of prosocial influence exclusive of potential interindividual or age differences in nonverbal reasoning abilities, we had participants take part in the Matrix Reasoning Item Bank (Chierchia et al., 2019). The task consists of a 3 × 3 matrix containing eight abstract shapes and a missing shape. Participants are asked to complete the pattern by clicking on the correct shape among four available alternatives. The proportion of correct choices was taken as a measure of nonverbal ability and thus used as an additional control. The task takes 8 min to complete.

Overall, the entire experimental session thus lasted around 35 min on average.

### Statistical analysis

The analysis includes four dependent variables. We first analyzed donations in Phase 1 because they are relevant to a distinct literature on age, gender, and economic behavior (reviewed by Sutter, Zoller, & Glätzle-Rützler, 2019) and because they could potentially affect social influence. In fact, social influence is generally proportional to the distance between one’s own baseline behavior (i.e., in this case, donations) and the decisions of others (Foulkes et al., 2018; Knoll et al., 2015; Moutoussis et al., 2016). In a follow-up analysis to participants’ first donations, we assessed whether there were age effects in the difference (delta) between participants’ first donations and the donations of other donors (Foulkes et al., 2018). We took the absolute value of this difference to obtain a more direct comparison of cases in which other donors gave more or less than participants (i.e., cases of prosocial influence vs. selfish influence).

Our central social-influence dependent variable was influence probability. To measure this, we first created a trial-level vector of 1s and 0s, where 0 indicated that no change in donation occurred between Phases 1 and 2 or that a change occurred but in the opposite direction of the observed donations, and 1 indicated that a change occurred in the direction of the observed donations. For a secondary dependent variable, to assess whether prosocial influence is associated with more deliberative or impulsive decision styles (Reiter et al., 2019), we investigated how response times (RTs) in Phase 2 varied as a function of whether or not participants were influenced and how this relation may change as a function of age and direction of influence. Finally, we analyzed influence magnitude, that is, the degree to which participants changed their donations in the direction of the observed donations between Phases 1 and 2. Specifically, following previous work (Foulkes et al., 2018; Knoll et al., 2017; Knoll et al., 2015; Reiter et al., 2019), we defined change as the amount donated in Phase 2 minus the amount donated in Phase 1. Then, all donation changes in the direction of the observed donations (i.e., conforming change) were transformed to positive (i.e., by taking the absolute value of change magnitude), whereas all changes in the opposite direction of the observed donations (i.e., anticonforming change) were taken as negative (i.e., by taking the absolute value of change and multiplying it by −1). Trials in which participants did not change their donations had a change value of 0. As main independent variables, the factors of the 2 × 3 × 3 design described above were used.

Raw trial-level data were modeled using generalized linear mixed models (GLMMs; Barr, Levy, Scheepers, & Tily, 2013) in the R programming environment (Version 3.4.1; R Core Team, 2017). Influence probability was modeled using the binomial distribution with logit link function. RTs lower than 250 ms (23 out of 7,906) were excluded from the analysis (Reiter et al., 2019). Remaining RTs were modeled on the log scale because this better approximated a normal distribution and additionally resulted in a lower Akaike information criterion (AIC) during model estimation. The three-way interaction among the main factors described above and all lower level interactions were included as fixed effects in all models. In the RT model only, we additionally included an influence term, a factor indicating whether or not the participant was influenced on the given trial (and all possible three-way interactions among this and the other factors of the model). In the influence-magnitude model only, following Foulkes and colleagues (2018), we additionally included the delta term (and all possible three-way interactions among this and the other factors of the model). Fixed effects for donations in Phase 1 included only age because the other factors did not apply. To obtain more parsimonious models, we progressively excluded nonsignificant higher level interactions via nested model comparison. All models clustered data by subject (i.e., as a random intercept) and additionally included maximal random slopes for the within-subjects factors (Barr et al., 2013) as random effects.

We modeled age as both categorical and continuous. When treating age as continuous, we first compared different curve-fitting regressions—linear, quadratic, and cubic (and combinations thereof) as well as inverse of age (1/age), logarithmic, and exponential (Luna, Garver, Urban, Lazar, & Sweeney, 2004)—in simpler models predicting the dependent variables of interest with the single independent variable (i.e., age alone). We then selected the trend or trends yielding the lowest AIC (to account for potential differences in the number of parameters) and forwarded this to the same models described above. For influence probability, the inverse of age had the lowest AIC. For the log of RTs, first donations, and influence magnitude, the lowest AIC was obtained by including linear, quadratic, and cubic components of age. For RTs and influence magnitude, but not first donations, the cubic component did not significantly contribute to the model fit and was thus discarded during model reduction. Polynomials were orthogonalized to eliminate multicollinearity. Main effects and interactions of the best-fitting models were inspected using omnibus Type III Wald χ^2^ tests. Planned and post hoc comparisons were performed using the *emmeans* package (Version 1.3.0; Lenth, Singmann, Love, Buerkner, & Herve, 2018) and Bonferroni-corrected for multiple comparisons.

We call the models described above *reference models* because they focused exclusively on the main variables of our experimental design. For each reference model, a number of additional control models probed the robustness of the findings to other potentially relevant factors. For example, given that adolescents and adults differ in a wide range of behaviors (Blakemore, 2018; Defoe et al., 2015; Reniers et al., 2017), these can introduce baseline differences in studies on social influence (given that social influence is frequently measured as a change in behavior relative to some baseline; Knoll et al., 2015; Moutoussis et al., 2016; Reiter et al., 2019). Therefore, to account for potential differences in baseline donations (either age-dependent differences or skewed patterns of baseline donations in general, such as participants who never donated anything) and the imbalance in delta that might have resulted from this, we added to one model a regressor for donations during Phase 1 and another for the delta term. A different control model was used to control for nonsocial-influence effects and thus to isolate influence effects that are not entirely explained by nonsocial processes. This control model focused on noncomputer trials only and included an additional regressor related to the degree of influence displayed by participants on computer trials. For influence probability, this regressor was the proportion of trials in which participants had been influenced in the computer condition. For influence magnitude, it was the mean influence magnitude displayed in the computer conditions. Finally, to control for response variability, we coded responses as 1 if participants conformed, as 0 if they did not change, and as −1 when they anticonformed. We then took the participant-level variance of this vector as a measure of response variability. We used this variance measure as a covariate to assess whether age-related decreases in conformity were still observed after controlling for age-related decreases in response variance.

For reasons of space, we provide the results of each of these models only for influence probability in the manuscript (Table 2), whereas the same control models for this and the other dependent variables can be found in Tables S10 through S17 in the Supplemental Material. Those supplemental tables also show the results of control models controlling for a number of other factors. For example, to account for potential gender differences in pubertal onset, one such control model controlled for gender and its interaction with age, and other models accounted for abstract-reasoning performance, group size, block order, and the guess of the token-pound exchange rate, among others. For RTs only, a control model additionally controlled for RTs of donations to the same charity during Phase 1 (for details on each control model, see Section 5 in the Supplemental Material).

**Table 2.** Estimates From the Influence-Probability Models

Variable	Reference model	Reference model + first donation	Reference model + delta	Reference model + response variance	Reference model (social-influence trials only) + computer influence
Intercept	−0.26 (0.2)	−0.28 (0.2)	−0.24 (0.2)	−1.6*** (0.23)	−0.51** (0.18)
Age group					
Midadolescents	−0.65* (0.25)	−0.65* (0.25)	−0.69** (0.26)	−0.36 (0.24)	−0.26 (0.23)
Adults	−1.05*** (0.27)	−1.1*** (0.27)	−1.19*** (0.27)	−0.72** (0.25)	−0.69** (0.24)
Source					
Adults	−0.05 (0.07)	−0.05 (0.07)	−0.05 (0.08)	−0.05 (0.07)	−0.05 (0.07)
Computer	−0.48*** (0.08)	−0.48*** (0.08)	−0.49*** (0.08)	−0.49*** (0.08)	
Direction of influence: selfish	−0.02 (0.28)	0.04 (0.28)	−0.04 (0.29)	−0.02 (0.27)	−0.03 (0.27)
Midadolescents × Selfish Influence	0.03 (0.37)	0.05 (0.37)	0.02 (0.38)	0.04 (0.36)	0.06 (0.36)
Adults × Selfish Influence	0.77^†^ (0.4)	0.77^†^ (0.4)	1.06* (0.41)	0.67^†^ (0.38)	0.61 (0.38)
First donation		−0.07 (0.06)			
Delta			0.3*** (0.03)		
Response variance				3.18*** (0.34)	
Computer influence					0.76*** (0.06)

Note: Values in parentheses are standard errors. For all control models, see Table S12 in the Supplemental Material available online.

† *p* < .10. *p < .05. **p < .01. ***p < .001.

In addition to these control models, we further ran a number of additional reduced models, which probed the robustness of results when various exclusion criteria were adopted (see Section 5). Among the latter, we assessed whether the omnibus effects of interest remained significant when we excluded participants who displayed skewed decision patterns (e.g., floor or ceiling effects) in the donations of Phase 1. At the request of a reviewer, we also ran an exploratory reduced model that included data from the adolescents only (adults were excluded; see Section 6 in the Supplemental Material).

All of the significant omnibus results reported below were robust to all such control models and reduced models unless otherwise noted (see Section 5). Data and scripts are available on OSF at https://osf.io/3e9s6/.

## Results

### Prosocial behavior at first donation

**Fig. 2.**
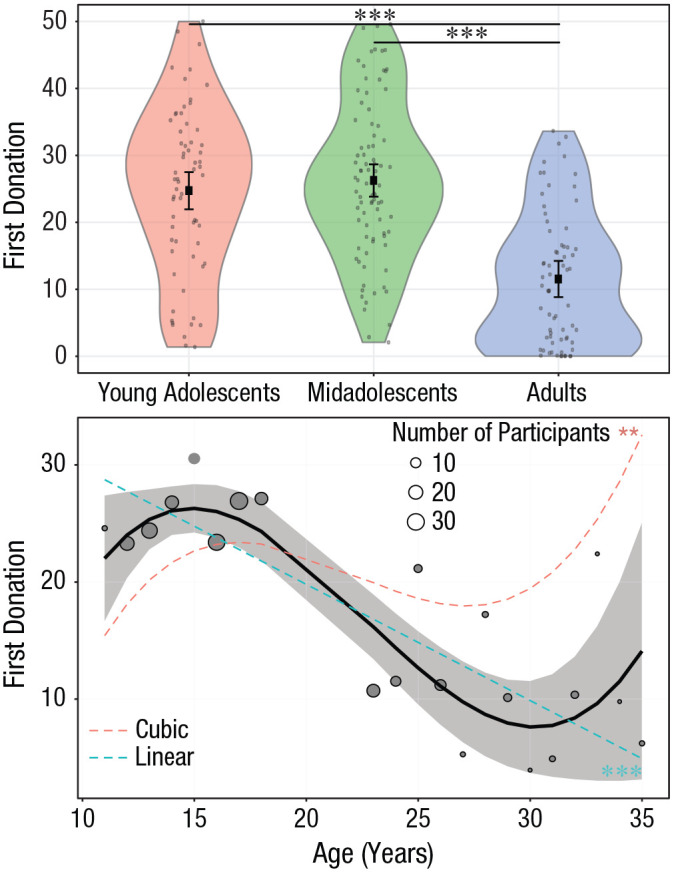
Effect of age on first donations (before participants observed what other people donated). In the top graph, age is treated as a categorical variable. Dots are individual participant means. The violin plots represent kernel probability density of the data at different values (randomly jittered across the *x*-axis). Within each age group, the black squares represent the fixed-effects estimates of first donations from the trial-level linear mixed model, and error bars show the corresponding 95% confidence intervals. Asterisks indicate significant differences between groups (*p* < .001, Bonferroni corrected). For statistics of all contrasts, see Table S1 in the Supplemental Material available online. In the bottom graph, age is treated as a continuous variable. Circles are grand means. Circle area is proportional to the number of participants; the key shows three examples for reference. The black line shows the overall polynomial trend as estimated by the trial-level generalized linear mixed model. The shaded area is the 95% confidence interval. The dashed, colored lines represent significant linear and cubic trends (**p < .01, ***p < .001).

A GLMM revealed a main effect of age group on first donations, χ^2^(2) = 72.13, *p* < .001 (Fig. 2, top panel; also see Fig. S3 and Section 7 in the Supplemental Material). Planned contrasts showed that adults donated less than both midadolescents and young adolescents (young adolescents – adults: contrast = 13.19, *SE* = 1.98, Bonferroni-corrected *p* [*p*_Bonf_] < .001; midadolescents – adults: contrast = 14.73, *SE* = 1.85, *p*_Bonf_ < .001; for all contrasts, see Table S1 in the Supplemental Material). The effect of age on first donations was also observed when age was modeled as a continuous variable: Donations linearly (and cubically) scaled with age—linear trend: χ^2^(2) = 55.84, *p* < .001; cubic trend: χ^2^(1) = 9.67, *p* = .002 (Fig. 2, bottom panel).

A control model additionally revealed a main effect of gender on first donations, χ^2^(1) = 7.87, *p* = .005, which did not interact with age, χ^2^(2) = 1.30, *p* = .521. Contrasts suggest that this was because female participants donated larger amounts than male participants (female – male: contrast = 5.66, *SE* = 1.52, *p*_Bonf_ < .001). This corroborates a frequently observed gender effect in prosocial behavior, discussed elsewhere (for reviews, see Sutter et al., 2019; Van der Graaff, Carlo, Crocetti, Koot, & Branje, 2018).

The age differences in baseline donations possibly led to differences in deltas between the age groups (i.e., the absolute difference between one’s own donations and the observed donations of others). Indeed, a mixed model showed a significant interaction between age group and direction on delta, χ^2^(2) = 40, *p* < .001, and post hoc contrasts suggested that, unsurprisingly, deltas under prosocial influence were greater for adults than for both adolescent groups (young adolescents – adults: contrast = −4.21, *SE* = 0.92, *p*_Bonf_ < .001; midadolescents – adults: contrast = −3.15, *SE* = 0.86, *p*_Bonf_ = .001), whereas deltas under selfish influence were greater for both adolescent groups than for adults (young adolescents – adults: contrast = 5.21, *SE* = 1.05, *p*_Bonf_ < .001; midadolescents – adults: contrast = 6.46, *SE* = 0.99, *p*_Bonf_ < .001). The low baseline donations of adults might also have led to within-group differences in deltas under prosocial and selfish influence. Indeed, contrasts within the same model suggested that deltas were skewed toward prosocial influence in adults (prosocial – selfish: contrast = 9.19, *SE* = 1.25, *p*_Bonf_ < .001) but were balanced in both young adolescents (prosocial – selfish: contrast = −0.23, *SE* = 1.28, *p*_Bonf_ = 1) and midadolescents (prosocial – selfish: contrast = −0.42, *SE* = 1.11, *p*_Bonf_ = 1).

Thirty-nine participants displayed a skewed pattern of first donations. For these participants, it was not possible to generate an equal number of prosocial and selfish influence trials: One participant always donated the maximum amount of 50 tokens, five participants always donated 0, nine participants were skewed toward the maximum (i.e., they donated the maximum amount in more than half of the trials), and 24 participants were skewed toward the minimum (i.e., they donated 0 in more than half of the trails). Our reduced models showed that all significant results reported in the study were robust to the exclusion of these participants.

### Prosocial-influence manipulation checks

After observing the amount of tokens given by other donors and being reminded of their own previous donation to a given charity, participants changed their donations in 43% of trials. In such cases, 76% of adjustments (2,624 of 3,433 trials) moved in the direction of the observed donations (i.e., consistent with a social-influence effect), whereas the complementary percentage (24%) moved in the opposite direction (i.e., anticonforming choices). Supplemental analyses further showed that age trends in anticonforming probability were entirely explained by interindividual differences in response variance, whereas age trends in conforming probability were not (see Section 8 in the Supplemental Material).

Exact binomial tests confirmed that these frequencies significantly differ from a uniform distribution (i.e., rejecting the null hypothesis that conforming and anticonforming adjustments occurred with equal probability; frequency of conforming adjustments = 0.76, 95% confidence interval, or CI = [0.75, 0.78], *p* < .001). This was also true when we inspected each of the 2 × 3 × 3 cells of our experimental design (all *p*_Bonf_s < .001) as well as for the computer conditions.

Similarly, inspecting the size of donation changes (averaged at the participant level), we found that *t* tests against 0 showed that the average change in donation was positive and statistically different from 0 (influence magnitude = 1.78, 95% CI = [1.48, 2.09], *p* < .001). This indicates that social-influence magnitude was on average larger when participants adjusted their donations toward as opposed to away from the donations they observed. With four exceptions, this too held (all *p*_Bonf_s < .035) for each cell of our experimental design. Three exceptions were in the computer condition: Influence magnitude in midadolescents was not significantly different from 0 under both prosocial and selfish influence, whereas the same held for young adolescents under selfish influence only. The fourth exception was in the adult group under selfish influence from teenagers. Taken together, these results suggest that participants’ second donations were reliably influenced by the donations they observed, in terms of both influence probability and influence magnitude.

Finally, we inspected the relation between social influence and one’s distance from the observed norms (i.e., Δ). For example, suppose participant *i* donated five tokens to a given charity at baseline and subsequently observed one of two norms: In one case, *i* observed other donors giving seven tokens to the same charity (thus, Δ = 2), whereas in another case, *i* observed that other donors gave 15 tokens to the same charity (thus, Δ = 10). It seems plausible that the second case may lead *i* to adjust his or her donation more than the first. In fact, previous studies have consistently reported that such a positive linear relation exists (e.g., Foulkes et al., 2018; Knoll et al., 2017; Knoll et al., 2015; Moutoussis et al., 2016). However, other studies have shown that there are boundary conditions to this linear social-influence effect (e.g., Shang & Croson, 2009). In particular, if deltas are very small or very large, participants may deem them irrelevant, and this may result in diminished social influence. If so, this may result in a quadratic relationship rather than a linear one. Our study adaptively capped the observed donations to avoid such extreme and irrelevant deltas. In addition, to assess whether this sufficed to isolate a linear relation between social influence and deltas, we fitted the social-influence variables (both influence probability and magnitude) to polynomial functions of delta (up to the fourth degree included) using mixed models and then compared these models using AICs. The model with the best fit was linear. This was true at the full-sample level, as well as for each of the six possible subsample combinations of age groups and direction of influence, for both influence probability and influence magnitude. More specifically, in each of these cases, the linear term was always significant and positive (influence magnitude: all slopes > 0.4, all *p*s < .001; influence probability: all slopes > 0.14, all *p*s < .050).

#### Influence probability

**Fig. 3.**
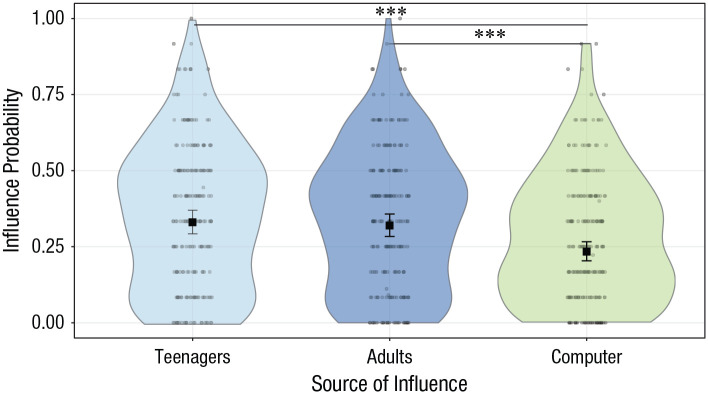
Effect of source of influence on influence probability. Dots are the frequencies of trials (%) in which participants changed their donations and conformed them with those of other donors. The violin plots represent kernel probability density of the data at different values (randomly jittered across the *x*-axis). Within each source type, the black squares represent the fixed-effects estimates of influence probability from the trial-level generalized (logistic) linear mixed model, and error bars show the corresponding 95% confidence intervals. Asterisks indicate significant differences between sources of influence (***p < .001, Bonferroni corrected). For statistics of all contrasts, see Table S2 in the Supplemental Material available online.

A GLMM revealed a significant main effect of source on influence probability, χ^2^(2) = 39.48, *p* < .001 (Fig. 3): the probability of changing one’s donation between Phase 1 and Phase 2 in the direction of the observed donation. Planned contrasts showed that participants were more likely to be influenced by other people than by the computer (teenagers – computer: contrast = 0.48, *SE* = 0.08, *p*_Bonf_ < .001; adults – computer: contrast = 0.43, *SE* = 0.08, *p*_Bonf_ < .001; for all contrasts, see Table S2 in the Supplemental Material). There was no significant interaction between source and any of the other factors (*p*s > .10).

**Fig. 4.**
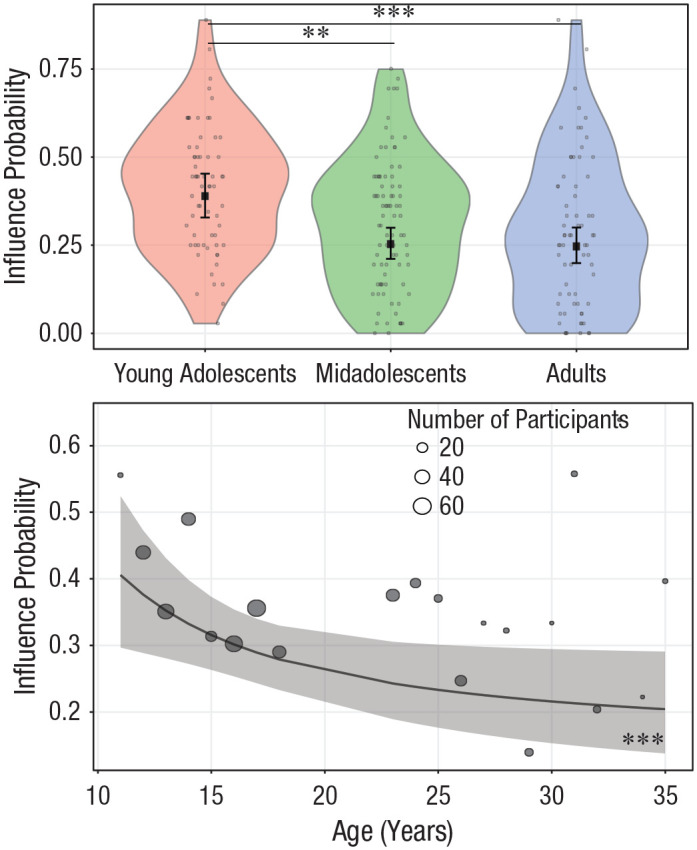
Effect of age on influence probability. In the top graph, age is treated as a categorical variable. Dots are the frequencies of trials (%) in which participants changed their donations and conformed them with those of other donors. The violin plots represent kernel probability density of the data at different values (randomly jittered across the *x*-axis). Within each age group, the black squares represent the fixed-effects estimates of influence probability from the trial-level generalized (logistic) linear mixed model, and error bars show the corresponding 95% confidence intervals. Asterisks indicate significant differences between groups (**p < .01, ***p < .001, Bonferroni corrected). For statistics of all contrasts, see Table S3 in the Supplemental Material available online. In the bottom graph, age is treated as a continuous variable. Circles are grand means of trials in which participants adjusted their donations to the observed donations. Circle area is proportional to the number of participants; the key shows three examples for reference. The black line shows the overall linear trend for the inverse of age as estimated by the generalized linear mixed model, and the shaded area is the 95% confidence interval. Asterisks indicate a significant trend (***p < .001).

The model also revealed a significant impact of age on influence probability, χ^2^(2) = 16.02, *p* < .001. Contrasts showed that young adolescents were more likely to be influenced than adults and midadolescents (young adolescents – midadolescents: contrast = 0.63, *SE* = 0.18, *p*_Bonf_ < .001; young adolescents – adults: contrast = 0.67, *SE* = 0.19, *p*_Bonf_ = .002; for all contrasts, see Table S3 in the Supplemental Material), whereas midadolescents and adults did not significantly differ in this respect (Fig. 4, top panel). The effect of age was also reliably observed when models used age as a continuous variable, χ^2^(1) = 20.31, *p* < .001: There was a linear relation between the inverse of age and influence probability (slope = 23.21, *SE* = 5.15, *p* < .001; Fig. 4, bottom panel).

**Fig. 5.**
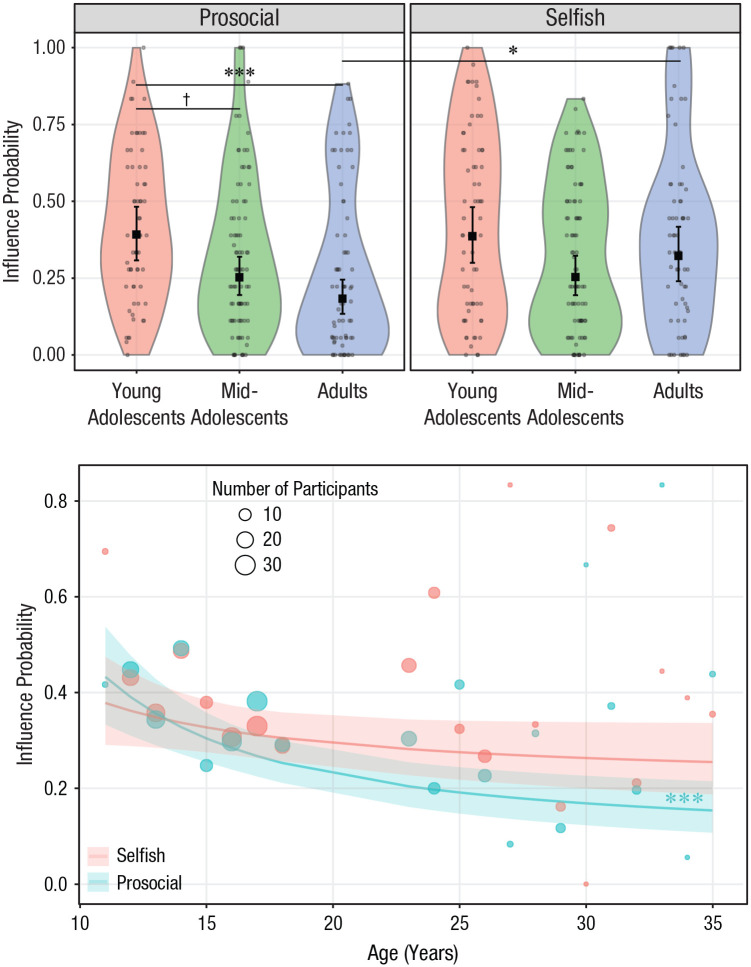
Interaction between age group and direction of influence (prosocial vs. selfish) on influence probability. In the top graph, influence probability is shown as a function of age (treated as a categorical variable), separately for the two directions of influence. Dots are the frequencies of trials (%) in which participants changed their donations and conformed them with those of other donors. The violin plots represent kernel probability density of the data at different values (randomly jittered across the *x*-axis). Within each age group, the black squares represent the fixed-effects estimates of influence probability from the trial-level generalized (logistic) linear mixed model, and error bars show the corresponding 95% confidence intervals. Symbols indicate significant and marginally significant differences between groups (^†^*p* < .10, *p < .05, ***p < .001, Bonferroni corrected). For statistics of all contrasts, see Tables S4a and S4b in the Supplemental Material available online. In the bottom graph, influence probability is shown as a function of age (treated as a continuous variable) and direction of influence. Circles are grand means of trials in which participants adjusted their donations to the observed donations. Circle area is proportional to the number of participants; the key shows three examples for reference. The colored lines shows the overall trends for the inverse of age as estimated by the generalized linear mixed model, and the shaded areas are 95% confidence intervals. Asterisks indicate a significant trend (***p < .001).

The effect of age was marginally modulated by the direction of influence, χ^2^(2) = 5.04, *p* = .080 (Fig. 5, top panel): Under prosocial influence, influence probability was higher for young adolescents than for adults (young adolescents – adults: contrast = 1.05, *SE* = 0.27, *p*_Bonf_ < .001; for all contrasts, see Table S4a in the Supplemental Material) and marginally higher than for midadolescents (young adolescents – midadolescents: contrast = 0.65, *SE* = 0.25, *p*_Bonf_ = .061), whereas this was not the case under selfish influence, where influence probability did not differ between age groups (all *p*_Bonf_s > .110). The interaction between age and direction was significant when the inverse of age was taken as a continuous predictor, χ^2^(1) = 3.95, *p* = .047 (Fig. 5, bottom panel). Contrasts suggested that the inverse of age decreased influence probability to a greater extent for prosocial influence, relative to selfish influence (prosocial – selfish: estimate = 13.87, *SE* = 6.98, *p* = .047). In line with this, post hoc analyses indicated that the linear trend of the inverse of age was present under prosocial influence (slope = 23.21, *SE* = 5.15, *p*_Bonf_ < .001) but not selfish influence (slope = 9.34, *SE* = 6.01, *p*_Bonf_ = .120).

To assess an effect of age on opportunistic conformity, we ran a separate set of within-age-group contrasts comparing influence probability under prosocial and selfish influence. These showed that adults were more likely to be influenced by other donors when others had given less than them, rather than more (prosocial – selfish: estimate = −0.75, *SE* = 0.28, *p*_Bonf_ = .025; for all contrasts, see Table S4b). This was not the case for the two adolescent age groups, whose donations were equally likely to conform to those of other donors, regardless of the direction of influence (*p*_Bonf_s = 1).

The significant omnibus effects reported above were robust to all control models. In particular, they remained significant when models controlled for participants’ first donations. Thus, even though first donations differed between age groups (Table 2), they did not cancel out the age differences in influence probability. Similarly, although the amount of influence exerted on participants (i.e., the Δ) also differed between age groups and robustly predicted influence probability, controlling for this did not cancel out the effects of age on influence probability (Table 2). It should also be noted that because deltas are positively associated with social influence (Foulkes et al., 2018; Knoll et al., 2015; Moutoussis et al., 2016), the particular pattern of age differences in deltas in our data would predict that adults would be more influenced than adolescents toward prosocial behavior and less influenced toward selfish behavior—the opposite pattern from that observed. Thus, it is highly unlikely that the age effects on social influence reported above were due to age differences in baseline donations or deltas.

Importantly, another control model showed that the significant age effects remained significant when models controlled for nonsocial influence (i.e., the proportion of trials in which participants had been influenced in the computer condition), suggesting that age effects of prosocial influence are not entirely explained by nonsocial anchoring effects. They were also robust when models controlled for response variability. In particular, even though response variance significantly contributed to the probability of conforming, age effects of conformity were not entirely explained by this (Table 2).

#### RTs

**Fig. 6.**
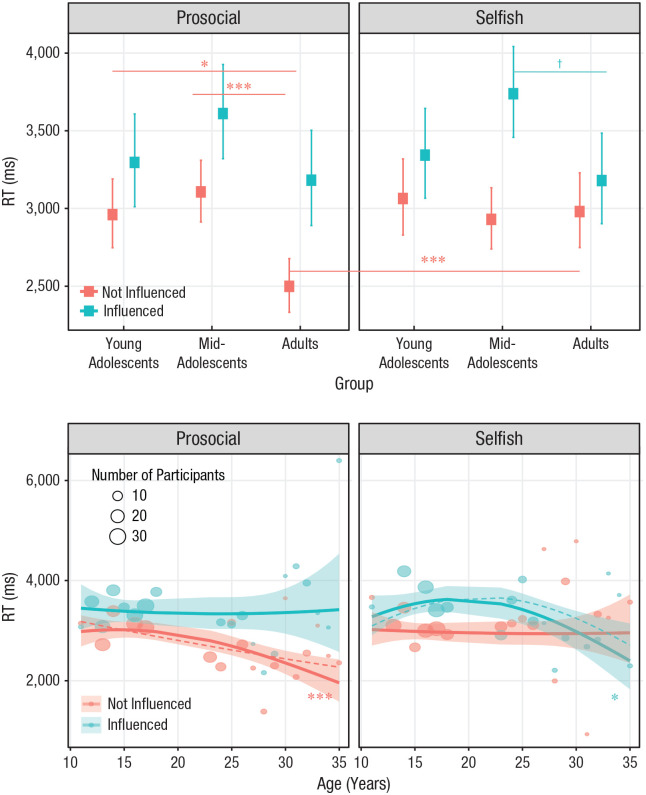
Interaction of age group, direction of influence (prosocial vs. selfish), and influence (influenced vs. not influenced) on response times (RTs). In the top graph, age is shown as a categorical variable. Colored squares show the fixed-effects estimates of RTs from the trial-level linear mixed model. Error bars are the corresponding 95% confidence intervals. Symbols indicate significant and marginally significant differences between and within groups (†*p* < .10, *p < .05, ***p < .001, Bonferroni corrected). For statistics of all contrasts, see Tables S5a and S5b in the Supplemental Material available online. In the bottom graph, age is shown as a continuous variable. Circles are grand medians of RTs. Circle area is proportional to the number of participants; the key shows three examples for reference. The lines show the overall polynomial trends of age as estimated by a trial-level generalized linear mixed model on the log of RTs (then back-transformed to the response scale). The shaded area is the 95% confidence interval. Asterisks indicate significant components of the trends (dashed lines; *p < .05, ***p < .001). For statistics of all trend contrasts, see Table S6 in the Supplemental Material.

A GLMM on the log of RTs during participants’ donations in Phase 2 revealed a main effect of the influence term (i.e., whether or not participants changed donation in the direction of the observed donation), χ^2^(1) = 8.08, *p* = .004: Contrasts showed that participants took longer to reach a decision when they adjusted their donations to the observed donation, relative to when they did not (influenced – not influenced: contrast = 0.15, *SE* = 0.02, *p* < .001). This effect was further modulated by age, as demonstrated by a significant three-way interaction among the influence term, participant age group, and the direction of influence, χ^2^(2) = 16.31, *p* < .001 (Fig. 6, top left panel). Contrasts suggested that adults took less time when resisting prosocial influence, relative to both young adolescents and midadolescents (young adolescents – adults: contrast = 0.17, *SE* = 0.05, *p*_Bonf_ = .013; midadolescents – adults: contrast = 0.22, *SE* = 0.05, *p*_Bonf_ < .001; for all contrasts, see Table S5a in the Supplemental Material). Furthermore, consistent with an opportunistic conformity effect, a separate set of within-age-group contrasts showed that adults were the only age group that took less time to resist prosocial influence than selfish influence (prosocial – selfish: contrast = −0.18, *SE* = 0.04, *p*_Bonf_ < .001; for all contrasts, see Table S5b in the Supplemental Material).

When we investigated age as a continuous variable, we found that two three-way interactions showed that both linear and quadratic components of age interacted with the direction of influence and the influence term—linear: χ^2^(1) = 16.32, *p* < .001; quadratic: χ^2^(1) = 13.78, *p* < .001 (Fig. 6, bottom panels). Contrasts demonstrated that under prosocial influence, RTs linearly decreased with age to a greater extent when participants were not influenced than when they were influenced (influenced – not influenced: contrast = 6.80, *SE* = 2.01, *p*_Bonf_ = .003), whereas under selfish influence, RTs were quadratically associated with age to a greater extent when participants were influenced as opposed to not influenced (influenced – not influenced: contrast = −5.21, *SE* = 1.96, *p*_Bonf_ = .031; for all contrasts, see Table S6 in the Supplemental Material). The dashed lines of Fig. 6 (bottom panel) highlight the components that interacted with the influence term: When participants were not influenced by more generous others, age was linearly associated with decreased RTs (slope = −7.33, *SE* = 1.81, *p*_Bonf_ < .001); when participants were influenced by more selfish others, age was quadratically associated with RTs, peaking between mid- and late adolescence (slope = −4.90, *SE* = 2.22, *p*_Bonf_ = .027).

#### Influence magnitude

**Fig. 7.**
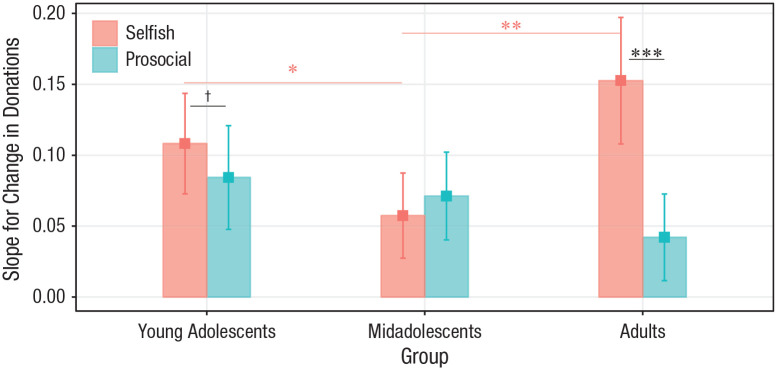
Interaction of age group, distance from norm, and direction of influence (prosocial vs. selfish) on influence magnitude. Slopes quantify the association between the distance from the norm and the subsequent magnitude of conforming donation change. Bars show fixed-effects estimates of slopes from the trial-level linear mixed model. Error bars are the corresponding 95% confidence intervals. Symbols indicate significant and marginally significant differences between groups (pink) and within groups (black; ^†^*p* < .10, *p < .05, **p < .01, ***p < .001, Bonferroni corrected). For statistics of all contrasts, see Table S8 in the Supplemental Material available online.

Influence magnitude was measured as the degree to which participants changed their donation in the direction of the observed donations. A linear mixed model showed a main effect of source, χ^2^(2) = 6.40, *p* = .041, suggesting that, as for influence probability, influence magnitude was adapted more to other people than to computers (teenagers – computer: contrast = 0.86, *SE* = 0.19, *p*_Bonf_ < .001; adults – computer: contrast = 0.64, *SE* = 0.15, *p*_Bonf_ < .001; for all contrasts, see Table S7 in the Supplemental Material). On the other hand, the model showed no main effect of age on influence magnitude, χ^2^(2) = 1.30, *p* = .521. Instead, it showed that age (and direction of influence) modulated the extent to which it affected subsequent adjustments (in a three-way interaction among delta, age group, and direction), χ^2^(2) = 16.56, *p* < .001 (Fig. 7). In other words, age and direction of influence affected slope differences in the positive relation between delta and influence magnitude. Specifically, contrasts showed that such slopes were greater when adults decreased their donations to comply with observed (selfish) norms, rather than increasing them (prosocial – selfish: slope = −0.12, *SE* = 0.03, *p*_Bonf_ < .001), whereas this distinction was only marginal or absent in both the young adolescent (prosocial – selfish: slope = −0.05, *SE* = 0.02, *p*_Bonf_ = .055) and midadolescent (prosocial – selfish: slope = 0.01, *SE* = 0.02, *p*_Bonf_ = 1) age groups. As for influence probability, this finding is consistent with the notion that adults but not adolescents display opportunistic conformity: changing their donations to a greater extent when other donors had given less than them, compared with when they had given more. Moreover, contrasts comparing the slopes between age groups showed that, under selfish influence, slopes were smallest for midadolescents, relative to either of the other two age groups (young adolescents – midadolescents: slope = 0.07, *SE* = 0.03, *p*_Bonf_ = .042; midadolescents – adults: slope = −0.10, *SE* = 0.03, *p*_Bonf_ = .008; for other contrasts, see Table S8 in the Supplemental Material), whereas slopes did not differ between age groups under prosocial influence (*p*_Bonf_s = 1). The model also revealed a marginal three-way interaction among age group, direction, and source, χ^2^(4) = 9.26, *p* = .055, because of adults’ higher susceptibility to being influenced by a computer under selfish influence, relative to other age groups. However, this effect broke down, χ^2^(4) = 4.27, *p* = .370, when we removed extreme values (< 1% of the data) from the model and thus will not be discussed further.

Similar effects were observed when age was used as a continuous variable. A linear mixed model showed a reliable main effect of source, χ^2^(2) = 26.74, *p* < .001, and a significant trend for the delta term of the model, χ^2^(1) = 36.39, *p* < .001: Participants were more influenced by other people (i.e., either teenagers or adults) than by the random computer (teenagers – computer: contrast = 0.90, *SE* = 0.19, *p*_Bonf_ < .001; adults – computer: contrast = 0.64, *SE* = 0.15, *p*_Bonf_ < .001; for all contrasts, see Table S9 in the Supplemental Material), and increasing distance from the norms (i.e., the Δ) predicted increased magnitude of donation adjustment (estimate = 0.85, *SE* = 0.14, *p* < .001). As for the categorical analysis of age, there was no linear effect of age on influence magnitude, χ^2^(1) = 0, *p* = .947, but there was a three-way interaction among delta, direction of influence, and age (both linear and quadratic components of age), linear: χ^2^(1) = 8.74, *p* = .003; quadratic: χ^2^(1) = 5.49, *p* = .019. Post hoc analyses suggested that the linear but not quadratic component of age marginally interacted with deltas under prosocial influence, χ^2^(1) = 3.80, *p* = .051: The linear component of age marginally decreased the effect of deltas on change in donations under prosocial influence (slope = −20.62, *SE* = 10.58, *p* = .053), whereas it increased it under selfish influence (slope = 44.22, *SE* = 18.70, *p* = .019), hence the interaction. On the other hand, the quadratic component of age was marginally associated with influence magnitude under selfish influence (slope = 32.96, *SE* = 18.21, *p* = .072) but not under prosocial influence (slope = −1.11, *SE* = 10.60, *p* = .917).

## Discussion

The current study showed that the probability of social influence decreased between early adolescence and adulthood, independently of the prosocial or selfish direction of influence. This age-dependent social-influence effect might have been due to adolescents being more uncertain than adults about their decisions and thus relying more on other donors to inform their choices (e.g., Moutoussis et al., 2016) or being motivated by a greater need to fit in with others. Our finding that heightened social influence was associated with increased RTs (also see Reiter et al., 2019) argues against the notion that heightened social influence is due to impulsive or reactive decision-making.

In addition, we found that participants of all ages were equally influenced by peers and nonpeers. Notably, the peer-matching procedure used in this study (showing adolescents how much other adolescents had donated, as opposed to how much adults had donated) was the same as the one used in three previous studies on risk perception or risky decisions, as well as in the study by Foulkes and colleagues (2018), which focused on hypothetical prosocial behavior. Despite employing the same peer manipulation, the studies involving risk showed that adolescents are more influenced by other teenagers than by adults (Knoll et al., 2017; Knoll et al., 2015; Reiter et al., 2019), whereas here, as well as in the other study on prosocial influence (Foulkes et al., 2018), adolescents were equally influenced by teenagers and by adults. These results suggest that peer influence during adolescence (namely, heightened susceptibility to social influence for peers relative to nonpeers) is domain dependent and that peer influence might play a greater role in the domain of risk than prosocial behavior.

Our results showed that, even if decisions were costly, and even if participants were explicitly reminded (at the single-trial level) that some of the numbers they were observing were generated by a random computer, participants still aligned their decisions with those numbers. This corroborates the notion that anchoring effects are highly resistant, even to explicit reminders of their irrelevance (Wilson et al., 1996). Importantly, however, our control condition also allowed us to detect variance in social influence that was not explained by nonsocial influence, in that participants were more influenced by other people than by the computer. In fact, when focusing on social-influence trials (i.e., not computer trials), we still observed the reported effects of age on social influence, also controlling for the nonsocial influence. This suggests that social-influence effects are not entirely explained by nonsocial processes.

Finally, opportunistic conformity (the tendency to conform with selfish norms more than prosocial norms) has been observed in a number of previous studies in adults (Charness et al., 2019; Croson & Shang, 2008; Dimant, 2019) as well as in the adult sample analyzed here (see Section 9 in the Supplemental Material). However, we found that midadolescents displayed no signs of a directional asymmetry in social influence. This age effect of opportunistic conformity was unanticipated and would need to be replicated. That said, the finding fits with those of previous studies in this area. For example, recent reviews have suggested that prosocial preferences plateau or peak during adolescence (Sutter et al., 2019; Van der Graaff et al., 2018), and prosocial preferences have been suggested to modulate opportunistic conformity (Wei et al., 2016). In line with this, our results showed that baseline donations, which are related to prosocial preferences (Böckler et al., 2016), were greater in adolescents relative to adults and that adolescents simultaneously displayed reduced opportunistic conformity. In addition, negative affect is reported to be heightened in midadolescents relative to young adolescents (Larson, Moneta, Richards, & Wilson, 2002), and self-conscious emotions peak during mid to late adolescence (Somerville et al., 2013). This might amplify negative feelings associated with selfish-norm compliance. For example, it could heighten guilt aversion, which is a frequent motive for prosocial behavior in adults (Battigalli & Dufwenberg, 2007), children (Hoffman, 1998), and adolescents (Roos, Hodges, & Salmivalli, 2014). We thus speculate that potential age differences in prosocial preferences, coupled with a heightened sensitivity toward guilt, may contribute to a more unbiased weighting of prosocial and selfish influence in midadolescents.

## Limitations

Because of restricted logistic control in school recruitment and testing, adolescent participants were tested in different experimental settings from adults: Their testing environment was more familiar (given that they were tested in their own schools), and they were tested in larger groups than adults. We cannot exclude that such different experimental settings may partly explain the differences observed between adolescents and adults. For example, the larger groups in which adolescents took part may have increased the social salience of the stimuli used in our task. Importantly, however, our control analyses showed that the probability of social influence (and the magnitude of selfish influence) also differed between young adolescents and midadolescents, even in the absence of such differences in experimental settings. This suggests that even if experimental settings partly modulated the results, they are not the only mechanism at play. It would be important in future studies to match testing group size and experimental settings.

Second, our study did not control for age differences in the utility of money. Previous studies have suggested that the value of money could decline with age (e.g., Fehr, Glätzle-Rützler, & Sutter, 2013), and if this extended to our participants, it is unlikely to explain our results: If the value of money declines with age and this was the sole factor driving the results (i.e., no age differences in willingness to conform), younger individuals would be less susceptible to (costly) prosocial influence, whereas our results showed the opposite. However, our findings are consistent with the opposite pattern, namely, that adolescents are less incentivized by money than adults. This would fit with the general framework of our proposal that adolescence is a period of social reorientation, during which social concerns (e.g., to fit in or learn from other people) might crowd out other factors such as monetary concerns (Gneezy, Meier, & Rey-Biel, 2011). In future studies, researchers should aim to control for this by assessing a task-independent measure of the perceived value of money at different ages.

## Conclusion

Our study suggests that heightened social influence during adolescence is not only a source of vulnerability but also one of opportunity (Van Hoorn et al., 2016), one in which heightened social concerns could be harnessed to modulate prosocial behavior. For example, one such intervention reported that public endorsement of anticonflict (e.g., antibullying) values by referent students reduced reports of school conflict by around 25% in 1 year, relative to control schools (Paluck, Shepherd, & Aronow, 2016). Finally, we found that for both adolescents and young adults, social anchors are more effective at modulating prosocial behavior than nonsocial anchors. This provides novel insight into the notion that social-norm-based interventions are a particularly effective device in promoting cooperation in the field (Kraft-Todd et al., 2015).

## Supplemental Material

sj-docx-1-pss-10.1177_0956797620957625 – Supplemental material for Prosocial Influence and Opportunistic Conformity in Adolescents and Young Adults Click here for additional data file. Supplemental material, sj-docx-1-pss-10.1177_0956797620957625 for Prosocial Influence and Opportunistic Conformity in Adolescents and Young Adults by Gabriele Chierchia, Blanca Piera Pi-Sunyer and Sarah-Jayne Blakemore in Psychological Science

## Transparency

*Action Editor:* Erika E. Forbes

*Editor:* D. Stephen Lindsay

*Author Contributions*

S.-J. Blakemore wrote the initial grant application. S.-J. Blakemore and G. Chierchia designed the study. G. Chierchia and B. Piera Pi-Sunyer conducted testing and data collection. G. Chierchia and B. Piera Pi-Sunyer analyzed and interpreted the data under the supervision of S.-J. Blakemore. All the authors contributed to the writing of the manuscript and approved the final manuscript for submission.

**Open Practices:** Deidentified data, stimuli, and scripts have been made publicly available via OSF and can be accessed at https://osf.io/3e9s6. The design and analysis plans for the study were not preregistered. This article has received the badges for Open Data and Open Materials. More information about the Open Practices badges can be found at http://www.psychologicalscience.org/publications/badges.

## References

[bibr1-0956797620957625] Anwyl-Irvine A. L. Massonnié J. Flitton A. Kirkham N. Evershed J. K. (2020). Gorilla in our midst: An online behavioral experiment builder. Behavior Research Methods, 52, 388–407. 31016684 10.3758/s13428-019-01237-x PMC7005094

[bibr2-0956797620957625] Barr D. Levy R. Scheepers C. Tily H. (2013). Random effects structure for confirmatory hypothesis testing: Keep it maximal. Journal of Memory and Language, 68, 255–278. doi:10.1016/j.jml.2012.11.001 PMC3881361 24403724

[bibr3-0956797620957625] Battigalli P. Dufwenberg M. (2007). Guilt in games. American Economic Review, 97, 170–176. doi:10.1257/aer.97.2.170

[bibr4-0956797620957625] Berthier J. M. Semple S. (2018). Observing grooming promotes affiliation in Barbary macaques. Proceedings of the Royal Society B: Biological Sciences, 285(1893), Article 20181964. doi:10.1098/rspb.2018.1964 PMC6304063 30963904

[bibr5-0956797620957625] Blakemore S.-J. (2018). Avoiding social risk in adolescence. Current Directions in Psychological Science, 27, 116–122. doi:10.1177/0963721417738144

[bibr6-0956797620957625] Böckler A. Tusche A. Singer T. (2016). The structure of human prosociality differentiating altruistically motivated, norm motivated, strategically motivated, and self-reported prosocial behavior. Social Psychological and Personality Science, 7, 530–541.

[bibr7-0956797620957625] Charness G. Naef M. Sontuoso A. (2019). Opportunistic conformism. Journal of Economic Theory, 180, 100–134. doi:10.1016/J.JET.2018.12.003

[bibr8-0956797620957625] Chierchia G. Fuhrmann D. Knoll L. J. Piera Pi-Sunyer B. Sakhardande A. L. Blakemore S.-J. (2019). The matrix reasoning item bank (MaRs-IB): Novel, open-access abstract reasoning items for adolescents and adults. Royal Society Open Science, 6, Article 190232. doi:10.1098/rsos.190232 PMC6837216 31824684

[bibr9-0956797620957625] Choukas-Bradley S. Giletta M. Cohen G. L. Prinstein M. J. (2015). Peer influence, peer status, and prosocial behavior: An experimental investigation of peer socialization of adolescents’ intentions to volunteer. Journal of Youth and Adolescence, 44, 2197–2210. doi:10.1007/s10964-015-0373-2 26525387 PMC5985442

[bibr10-0956797620957625] Croson R. Shang J. (2008). The impact of downward social information on contribution decisions. Experimental Economics, 11, 221–233. doi:10.1007/s10683-007-9191-z

[bibr11-0956797620957625] Defoe I. N. Dubas J. S. Figner B. van Aken M. A. G. (2015). A meta-analysis on age differences in risky decision making: Adolescents versus children and adults. Psychological Bulletin, 141, 48–84. doi:10.1037/a0038088 25365761

[bibr12-0956797620957625] Dimant E. (2019). Contagion of pro- and anti-social behavior among peers and the role of social proximity. Journal of Economic Psychology, 73, 66–88. doi:10.1016/j.joep.2019.04.009

[bibr13-0956797620957625] Fehr E. Glätzle-Rützler D. Sutter M. (2013). The development of egalitarianism, altruism, spite and parochialism in childhood and adolescence. European Economic Review, 64, 369–383. doi:10.1016/J.EUROECOREV.2013.09.006

[bibr14-0956797620957625] Foulkes L. Leung J. T. Fuhrmann D. Knoll L. J. Blakemore S.-J. (2018). Age differences in the prosocial influence effect. Developmental Science, 21, Article e12666. doi:10.1111/desc.12666 PMC6221149 29658168

[bibr15-0956797620957625] Geier C. F. Terwilliger R. Teslovich T. Velanova K. Luna B. (2010). Immaturities in reward processing and its influence on inhibitory control in adolescence. Cerebral Cortex, 20, 1613–1629. doi:10.1093/cercor/bhp225 19875675 PMC2882823

[bibr16-0956797620957625] Gneezy U. Meier S. Rey-Biel P. (2011). When and why incentives (don’t) work to modify behavior. Journal of Economic Perspectives, 25, 191–210. doi:10.1257/jep.25.4.191

[bibr17-0956797620957625] Hoffman M. L. (1998). Varieties of empathy-based guilt. In Bybee J. (Ed.), Guilt and children (pp. 91–112). San Diego, CA: Academic Press. doi:10.1016/B978-012148610-5/50005-9

[bibr18-0956797620957625] Knoll L. J. Leung J. T. Foulkes L. Blakemore S.-J. (2017). Age-related differences in social influence on risk perception depend on the direction of influence. Journal of Adolescence, 60, 53–63. doi:10.1016/J.ADOLESCENCE.2017.07.002 28753485 PMC5614112

[bibr19-0956797620957625] Knoll L. J. Magis-Weinberg L. Speekenbrink M. Blakemore S.-J. (2015). Social influence on risk perception during adolescence. Psychological Science, 26, 583–592. doi:10.1177/0956797615569578 25810453 PMC4426139

[bibr20-0956797620957625] Kraft-Todd G. Yoeli E. Bhanot S. Rand D. (2015). Promoting cooperation in the field. Current Opinion in Behavioral Sciences, 3, 96–101. doi:10.1016/J.COBEHA.2015.02.006

[bibr21-0956797620957625] Larson R. W. Moneta G. Richards M. H. Wilson S. (2002). Continuity, stability, and change in daily emotional experience across adolescence. Child Development, 73, 1151–1165. doi:10.1111/1467-8624.00464 12146740

[bibr22-0956797620957625] Lenth R. Singmann H. Love J. Buerkner P. Herve M. (2018). Emmeans: Estimated marginal means, aka least-squares means (R package Version 1.3.0) [Computer software]. Retrieved from https://cran.r-project.org/web/packages/emmeans/index.html

[bibr23-0956797620957625] Luna B. Garver K. E. Urban T. A. Lazar N. A. Sweeney J. A. (2004). Maturation of cognitive processes from late childhood to adulthood. Child Development, 75, 1357–1372. doi:10.1111/j.1467-8624.2004.00745.x 15369519

[bibr24-0956797620957625] Moutoussis M. Dolan R. J. Dayan P. (2016). How people use social information to find out what to want in the paradigmatic case of inter-temporal preferences. PLOS Computational Biology, 12(7), Article e1004965. doi:10.1371/journal.pcbi.1004965 PMC4957786 27447491

[bibr25-0956797620957625] Nook E. C. Ong D. C. Morelli S. A. Mitchell J. P. Zaki J. (2016). Prosocial conformity. Personality and Social Psychology Bulletin, 42, 1045–1062. doi:10.1177/0146167216649932 27229679

[bibr26-0956797620957625] Paluck E. L. Shepherd H. Aronow P. M. (2016). Changing climates of conflict: A social network experiment in 56 schools. Proceedings of the National Academy of Sciences, USA, 113, 566–571. doi:10.1073/pnas.1514483113 PMC4725542 26729884

[bibr27-0956797620957625] R Core Team. (2017). R: A language and environment for statistical computing. Retrieved from http://www.R-project.org

[bibr28-0956797620957625] Reiter A. M. F. Suzuki S. O’Doherty J. P. Li S.-C. Eppinger B. (2019). Risk contagion by peers affects learning and decision-making in adolescents. Journal of Experimental Psychology: General, 148, 1494–1504. doi:10.1037/xge0000512 30667261

[bibr29-0956797620957625] Reniers R. L. E. P. Beavan A. Keogan L. Furneaux A. Mayhew S. Wood S. J. (2017). Is it all in the reward? Peers influence risk-taking behaviour in young adulthood. British Journal of Psychology, 108, 276–295. doi:10.1111/bjop.12195 26990740

[bibr30-0956797620957625] Roos S. Hodges E. V. E. Salmivalli C. (2014). Do guilt- and shame-proneness differentially predict prosocial, aggressive, and withdrawn behaviors during early adolescence? Developmental Psychology, 50, 941–946. doi:10.1037/a0033904 23895166

[bibr31-0956797620957625] Schmidt M. F. H. Tomasello M. (2012). Young children enforce social norms. Current Directions in Psychological Science, 21, 232–236. doi:10.1177/0963721412448659

[bibr32-0956797620957625] Shang J. Croson R. (2009). A field experiment in charitable contribution: The impact of social information on the voluntary provision of public goods. The Economic Journal, 119, 1422–1439. doi:10.1111/j.1468-0297.2009.02267.x

[bibr33-0956797620957625] Somerville L. H. Jones R. M. Ruberry E. J. Dyke J. P. Glover G. Casey B. J. (2013). The medial prefrontal cortex and the emergence of self-conscious emotion in adolescence. Psychological Science, 24, 1554–1562. doi:10.1177/0956797613475633 23804962 PMC3742683

[bibr34-0956797620957625] Steinberg L. (2008). A social neuroscience perspective on adolescent risk-taking. Developmental Review, 28, 78–106. doi:10.1016/J.DR.2007.08.002 18509515 PMC2396566

[bibr35-0956797620957625] Sutter M. Zoller C. Glätzle-Rützler D. (2019). Economic behavior of children and adolescents–A first survey of experimental economics results. European Economic Review, 111, 98–121. doi:10.1016/J.EUROECOREV.2018.09.004

[bibr36-0956797620957625] Van der Graaff J. Carlo G. Crocetti E. Koot H. M. Branje S. (2018). Prosocial behavior in adolescence: Gender differences in development and links with empathy. Journal of Youth and Adolescence, 47, 1086–1099. doi:10.1007/s10964-017-0786-1 29185207 PMC5878203

[bibr37-0956797620957625] van Goethem A. A. J. van Hoof A. van Aken M. A. G. Orobio de Castro B. Raaijmakers Q. A. W. (2014). Socialising adolescent volunteering: How important are parents and friends? Age dependent effects of parents and friends on adolescents’ volunteering behaviours. Journal of Applied Developmental Psychology, 35, 94–101. doi:10.1016/J.APPDEV.2013.12.003

[bibr38-0956797620957625] Van Hoorn J. Van Dijk E. Güroğlu B. Crone E. A. (2016). Neural correlates of prosocial peer influence on public goods game donations during adolescence. Social Cognitive and Affective Neuroscience, 11, 923–933. doi:10.1093/scan/nsw013 26865424 PMC4884312

[bibr39-0956797620957625] Wei Z. Zhao Z. Zheng Y. (2016). Moderating effects of social value orientation on the effect of social influence in prosocial decisions. Frontiers in Psychology, 7, Article 952. doi:10.3389/fpsyg.2016.00952 PMC4914595 27445917

[bibr40-0956797620957625] Wilson T. D. Houston C. E. Etling K. M. Brekke N. (1996). A new look at anchoring effects: Basic anchoring and its antecedents. Journal of Experimental Psychology: General, 125, 387–402. 8945789 10.1037//0096-3445.125.4.387

[bibr41-0956797620957625] Wolf L. K. Bazargani N. Kilford E. J. Dumontheil I. Blakemore S.-J. (2015). The audience effect in adolescence depends on who’s looking over your shoulder. Journal of Adolescence, 43, 5–14. doi:10.1016/J.ADOLESCENCE.2015.05.003 26043167 PMC4533226

