# Supplementary material for: Prosocial Influence and Opportunistic Conformity in Adolescents and Young Adults
Source: Psychol Sci. 2020 Nov 23;31(12):1585–601. doi: 10.1177/0956797620957625 (PMC7734552; doi:10.1177/0956797620957625)
Supplement: sj-docx-1-pss-10.1177_0956797620957625 – Supplemental material for Prosocial Influence and Opportunistic Conformity in Adolescents and Young Adults [file sj-docx-1-pss-10.1177_0956797620957625.docx]

**Supplemental Material**

**Supplemental information**

*Section 1. Supplemental information 1: participants with special educational needs*

Six participants in the adolescent sample (2 YA, 4 MA) had special needs (4 with mild dyslexia, 1 slow processing, 1 with borderline ADHD). The schools’ SENCO (Special Educational Needs Coordinator) deemed them capable of understanding the study instructions. Their data is thus included in the main analysis of our study (and only subsequently excluded as a control).

*Section 2. Supplemental information 2: incentivisation*

Participants were informed that they would earn a fixed payment of £7.50 for participating in the study and that, in addition to this, they could earn a bonus payment depending on the decisions they made during the task. They were told they did not have to worry about how to divide the tokens between charities as they would be given 50 new tokens for each charity. Following standard behavioural economics protocol, participants were informed that, at the end of the session, a single trial would be randomly selected and paid according to actual decisions. This occurred as stated: at the end of the task, participants viewed a ‘Payment screen’ showing the randomly selected charity, the number of tokens they had donated to that charity, the token-pound conversion rate, and thus the payment to themselves and to the charity (converted into pounds). Participants received the corresponding payment at the end of the experimental session.

Since the value of money likely changes with age, participants were not informed of the exact token conversion rate beforehand, and were only informed that tokens were worth money (van den Bos, van Dijk, Westenberg, Rombouts, & Crone, 2011). Before the payment screen, participants were probed on how much they thought each token was worth. They provided this information by means of a visual analogue scale ranging between 0 to 100 pence. This allowed participants to guess that the maximum bonus (i.e., if no token is donated) could range anywhere between 0 to 50 pounds (i.e., if they provided a rating of 0 or 100 pence respectively). The value of each of each token was actually 5 pence. Thus, in addition to the fixed show up fee of £7.50, participants could earn a decision-dependent bonus payment that varied between £0 and £2.50. We removed the true value of tokens away from the center of the scale to better individuate participants that thought this question through and thus did not default to the centre of the scale. The mean and confidence interval of guesses (mean = 33.72, 95% CI [29.52 37.92]) was in fact far removed from the middle of the scale (i.e, 50). Moreover, we individuated no age differences in the guess of conversion rate, both when using age as a categorical predictor (F_(2,217)_ = 1.61, p = 0.203) (Fig. S1), as well as when inspecting linear, quadratic and cubic trends of age (all p_s_ > 0.278). Nonetheless, we used this ‘guess of the conversion rate’ to control for any participant-level differences in payment expectations.

**Figure S1. No age differences in guess of exchange rate.** Points represent participant-level guesses (i.e., one per participant) of the token-pound exchange rate (on a continuous scale between 0 and 100 pence). Violin plots represent kernel probability density of the data at different values (randomly jittered across the x-axis). Within each age group, the black squares represent the estimated guesses based on linear regression, and error bars show the corresponding 95% confidence intervals.

*Section 3. Supplemental information 3: observed donations*

The observed donations were in reality determined by an adaptive algorithm, which customized them to each participant’s first donations. This algorithm aimed to balance the number of ‘prosocial influence’ trials in which others donated *more generously* than the participant, and ‘selfish influence’ trials in which others donated *more selfishly* than the participant.

To achieve this, the algorithm first sorted the charities based on how much participants donated to them in phase 1. It then assigned the lower half to the prosocial influence condition and the upper half to the selfish influence condition. In addition to balancing the number of prosocial and selfish influence trials, this was also done to increase the distance between participants’ first donations and observed donations. Specifically, the observed donation was a random number in the relevant interval: observed donation ∈ [(donation 1 + 1), 45] for prosocial influence trials, and observed donation ∈ [(donation 1 - 1), 5] for selfish influence trials. These intervals were capped at 45 and 5 in order to avoid implausible observed donations.

In a first step, the algorithm assigned an equal number of charities (namely half or 18 of the charities) to the Prosocial influence and Selfish influence conditions, and in a second step it randomly assigned an equal number of those charities (a third, or 6) to the Teenager, Adult or Computer conditions. The first of these two steps (counterbalancing the direction of influence factor) was inherently not possible for participants displaying highly skewed initial donations, as illustrated in Figure S2 below. It also occasionally failed due to a minor error in the algorithm. This is described below for researchers reanalyzing our data who might otherwise ask why some of the observed donations do not perfectly align with the algorithm described above.

Overall, 39 participants (out of 220) displayed skewed first donations: 1 participant (a young adolescent) always donated the maximum amount of 50 tokens, 5 participants (all adults) always donated 0, 9 were skewed towards the maximum (i.e., they donated the maximum amount in more than half of the trials) (3 young adolescents and 6 mid adolescents), and 24 were skewed towards the minimum (i.e., they donated 0 in more than half of the trails) (4 young adolescents, 2 mid adolescents and 18 adults). All significant results were robust to the exclusion of these participants.

The fault in the algorithm was due to its accidentally reading donation values of phase 1 as characters instead of numbers. This partially affected how the charities were sorted and thus how they were assigned to the prosocial and selfish influence conditions. For example, if the initial donations to 4 charities were 1, 3, 10 and 21, the algorithm would interpret the ascending order to be 1, 10, 3 and 21 (instead of the numeric order: 1, 3, 10, 21). Figure S2, panel D represents the trials of a participant for which this occurred. Fortunately, this problem only partially affected the intended outcome because, in most cases, ordering number between 0 and 50 as characters or numbers results in the same sequence. This also contributed to its missed detection during piloting. The error was detected roughly one fourth through data collection process and was corrected thereafter. Overall, less than 7% of trials was affected by the bug in a subset of 65 participants, mostly adults (n=31) or young adolescents (n=27).

Two control measures were taken to account for the possible impact of this error on the results (see Section 5 for additional information). First, analyses were run including and excluding the affected trials. Second, **Δ** (i.e., the difference between what participants donated and what others donated) were included as covariates in the statistical models. All the omnibus tests reported in the manuscript remained significant in such follow-up models, suggesting that the defect in the algorithm did not affect results in any meaningful way.


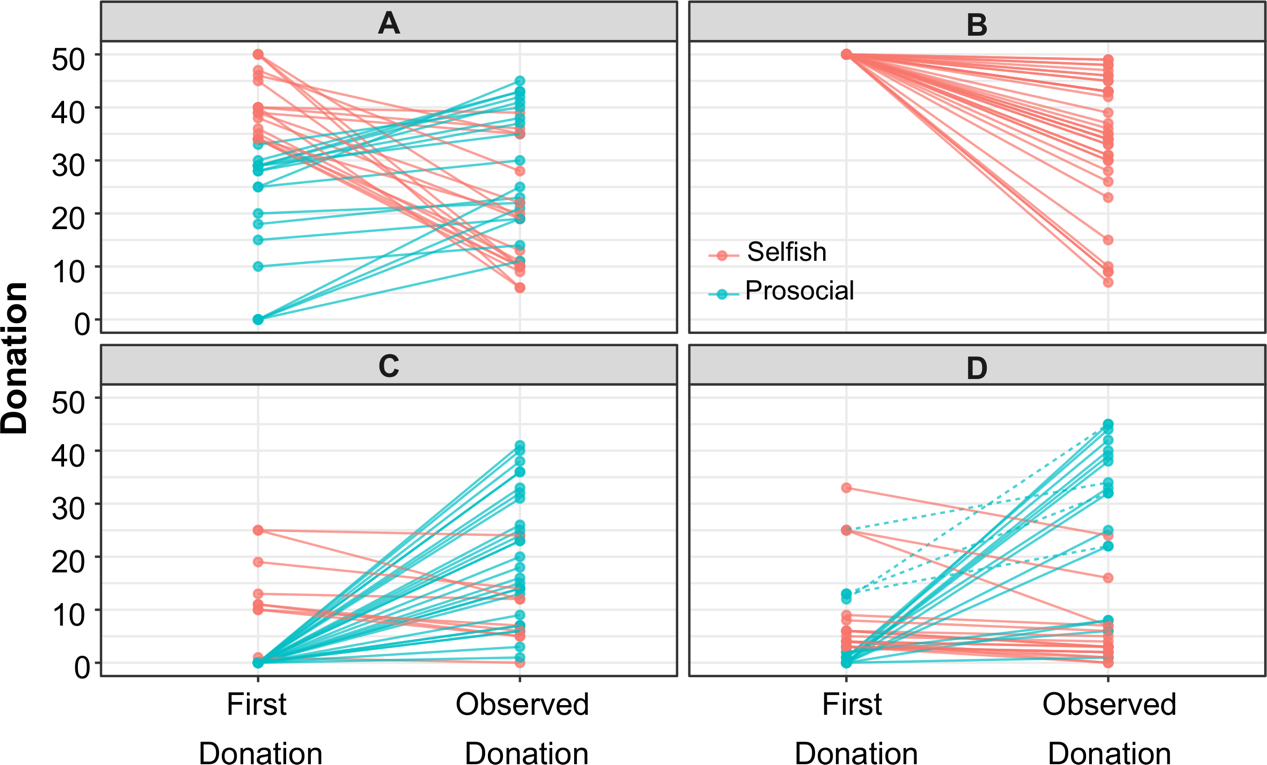


**Figure S2.** Initial donations (phase 1) and observed donations (phase 2) in four participants. Panel A: sufficiently spread initial donations allowed assigning an equal number of charities to prosocial and selfish influence trials. Panel B: a participant who always donated the maximum could only take part in selfish influence trials. Panel C: a participant who gave the minimum on more than half the trials would take part in more prosocial influence than selfish influence trials. Panel D: in less than 7% of trials, counterbalancing was not fully achieved because an error in the algorithm. In the case of this participant, this led some charities (dashed lines) to be erroneously assigned to the prosocial condition. Removing trials affected by this error did not qualitatively alter the results.

*Section 4. Supplemental information 4: stimuli*

In the prosocial influence task participants decided how many tokens to donate to 36 charities. To decorrelate effects from any particular charity content, for each participant, the 36 charities were randomly selected amongst a list of 120 possible charities. Each charity included a picture and a short sentence related to the charity’s mission. All pictures were taken from Google images and were labelled as free-for-reuse and modification. The pictures were cropped so as to have the same size and the sentences were of comparable length (mean number of characters = 47, SE = 1).

The charities were equally divided into one of 6 broad categories, thus amounting to 20 different charities for each category. Each participant was assigned a random subset of 6 charities per category. The categories were related to health issues, social issues, environmental issues, animal protection, education and one broader category related to war, poverty and natural disasters.

All stimuli are available online in the open science framework (<https://osf.io/3e9s6/>).

*Section 5. Supplemental information 5: control models*

Control models (CMs) probed the robustness of our findings to a number of potentially important factors, each added as a regressor to the main model presented in the manuscript: CM1) Gender, as well as its interaction with age (to control for possible effects of earlier pubertal onset in females); CM2) Guess exchange rate (z-scored) (see Section 2); CM3) Non-verbal reasoning (z-scored), as measured by the MaRs-IB; CM4) first donation; CM5) Δs, that is, the absolute difference between first donation and observed donation [obsDon – don1] (z-scored); CM6) Block order (i.e., whether participants started the Teenager, Adult or Computer block); CM7) Group size; CM8) For the reaction time model one, reaction times for the donations to the same charity during phase 1; CM9) To control for non-social influence effects, and thus isolate influence effects that may be uniquely ‘social’, this CM focused on non-computer trials only and added a regressor related to the degree of influence displayed by participants on computer trials. For influence probability, this additional regressor was the proportion of trials in which participants had been influenced in the computer condition. For influence magnitude, it was the mean influence magnitude displayed in the computer conditions. CM10) For the influence magnitude model only, we capped the dependent variable at Δ. CM11) To control for response variability we coded responses as 1 if participants conformed, as 0 if they did not change, and as -1 when they anti-conformed. We then took the participant-level variance of this vector as a measure of response variability. We used this variance measure as a covariate, to assess whether age-related decreases in conformity are still observed after controlling for age-related decreases in response variance. CM12) To further control for differences in expected exchange rates, all token-related regressors were multiplied by the participant-specific guesses of the exchange rate. For example, suppose that on a given trial, participant *i* and participant *j* observed that others had donated 5 tokens more than they did, and that both participants decided to increase their donations by the same amount (thus aligning their own donations to the observed donations). The influence magnitude analysis reported in the manuscript uses the number 5 as the dependent variable, since this is the amount by which these participants conformed (the magnitude of influence). Suppose now that *i* and *j* have different expectations with regard to the exchange rate: *i* believes a token is worth 5 pence, while *j* expects it to be worth 10. In this case, in terms of objective tokens these participants conformed to the same degree, however, in terms of subjective expectations, *j*’s conforming is more costly than *i*’s (i.e., since *j* believes tokens are worth more). To adjust for this, these control models assume that *i* conformed by 25 (i.e., 5 tokens multiplied by i’s expected exchange rate, in this example, 5 pence), while j conformed by 50 (i.e., 5 tokens multiplied by j’s expected exchange rate, that is, 10 pence). We applied this transformation to the single relevant dependent variable, namely, influence magnitude, as well to the Δs of all independent variables (see CM5 above). Analyses CM4-CM7 are recommended by previous literature (Reiter et al., 2018; Foulkes et al., 2018). CM8 to CM12 were run upon reviewer request. Results of the CM models are available in the supplemental material (Tables S10 – S17).

We also ran a number of reduced models (RMs), which adopted 6 potentially relevant exclusion criteria: RM1) participants who expressed suspicion of deception; RM2) participants with special educational needs (see Section 1 for additional information); RM3) trials affected by a defect in the script (see Section 3 for additional information); RM4) extreme values; RM5) participants displaying skewed donations during phase 1 (see Section 3 for additional information); and RM6) adult participants. RM6 was run upon reviewer request (see Section 6 for details).

A subset of the CM and RM models did not converge. We thus simplified the random effect structure of these models by removing the random slope for the ‘source of influence’ factor. This occurred for the IP model using age as a categorical variable (RM6), for the RT models using age as a categorical predictor (CM1, CM3, CM11, RM3) and as a continuous predictor (CM6), and for the influence magnitude models using age as categorical and continuous predictor (CM10, CM12, RM1, RM6). Significant omnibus tests were robust to all control models and exclusions, with the following exceptions: in the analyses of influence magnitude, the interaction between the quadratic trend of age, Δ and direction of influence (i.e., when using age as a continuous predictor) was no longer significant when controlling for the Δ adapted to the guess of the exchange rate (i.e., CM12). Also, age effects on RTs were no longer observed after excluding the adult group (i.e., RM6, see Section 6 for more information).

*Section 6. Supplemental information 6: adolescent participants only*

Adults displayed relatively lower donations than adolescents in phase 1 of the study. This resulted in greater distance from the observed donations (i.e. Δs) under prosocial influence, than under selfish influence. Upon reviewer request, we re-ran all analyses focussing on adolescent participants only (Tables S18 and S19). The results from these exploratory analyses are adjusted for multiple comparisons using Bonferroni correction, to account that existing models were run a second time.

In terms of influence probability, YA were more likely to be influenced than MA (*χ*^2^(1) = 7.99, *p*_Bonf_ = 0.009, contrast _YA – MA_ = 0.61, *p*_Bonf_ < 0.001) and the inverse of age was associated with a linear decrease in influence probability in this age range (*χ*^2^(1) = 10.46, *p*_Bonf_ = 0.002, slope = 25.28, SE = 7.82, *p*_Bonf_ = 0.002). These age effects did not interact with the direction of influence (age categorical: *χ*^2^(1) = 0.00, *p*_Bonf_  = 1; age continuous: *χ*^2^(1) = 0.45, , *p*_Bonf_ = 1, slope = 8.21, SE = 12.24, *p*_Bonf_ = 1). As per influence magnitude, we observed a three-way interaction between age, Δ and direction (*χ*^2^(1) = 5.42, *p*_Bonf_ = 0.040), due to MAs displaying less selfish influence than YAs (YA – MA selfish = 0.07, *p*_Bonf_ = 0.019), but not less prosocial influence (*p*_Bonf_ = 1). Similarly, linear (slope = 831.56, SE = 323.51, *p*_Bonf_ < 0.020) and quadratic (slope= 411.71, SE = 145.84, *p*_Bonf_ = 0.010) trends of age interacted with deltas and direction in predicting influence magnitude. Finally, we observed a social influence effect in RTs independent of age group (*χ*^2^(1) = 7.83, *p*_Bonf_ = 0.010): both young and mid adolescents took longer to reach a decision when they were influenced as opposed to when they were not (contrast_Influenced - Not influenced_ = 0.15, SE = 0.02, *p*_Bonf_ < 0.001), speaking against the notion that heightened social influence during adolescence is related to heightened impulsivity (Reiter, Suzuki, O’Doherty, Li, & Eppinger, 2019).

*Section 7. Supplemental information 7: Item-level donations in phase 1 by age group*


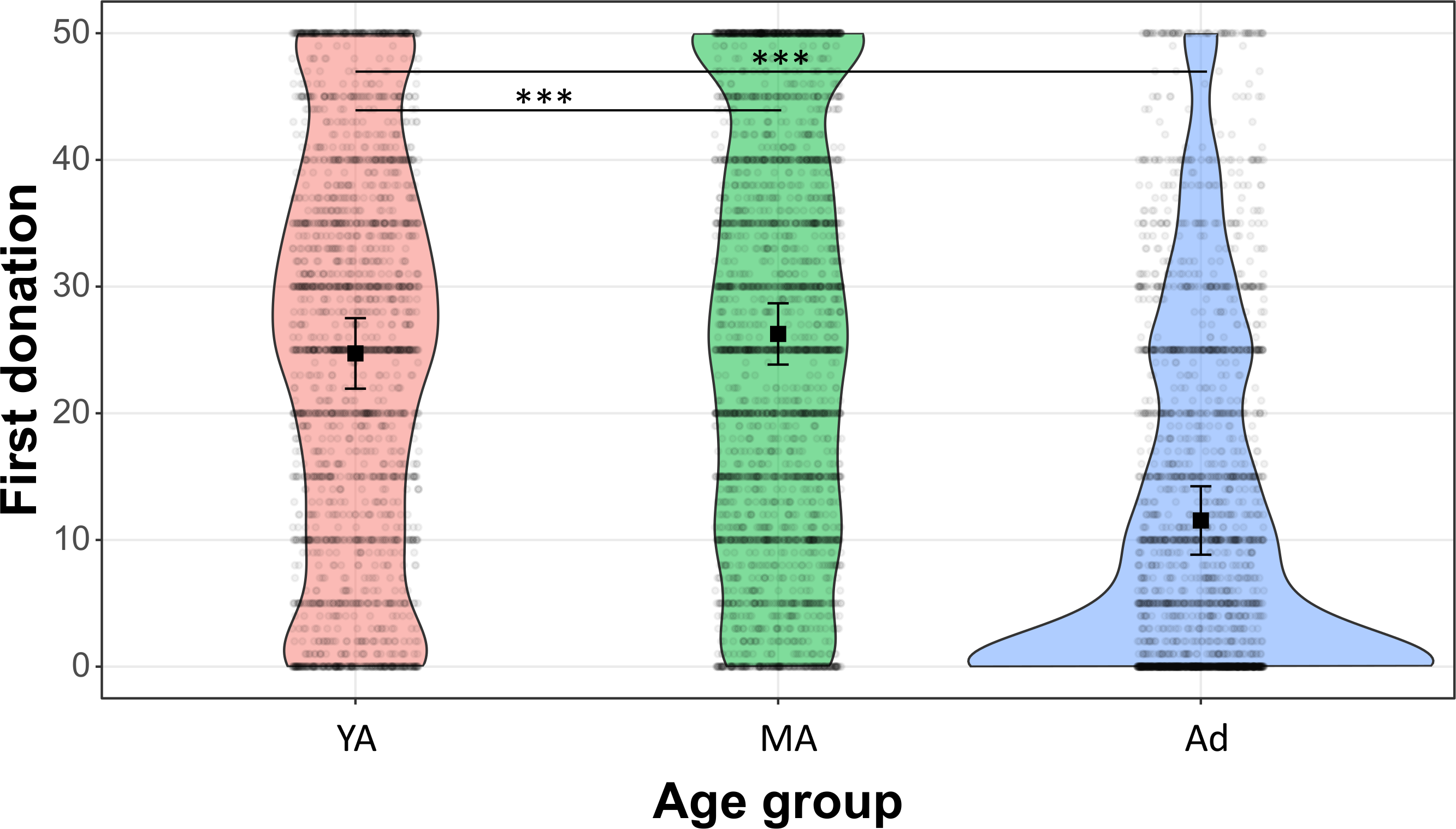


**Figure S3. Main effect of age group on donations in phase 1*.*** Dots are participants’ trial-level first donations (before participants observed what others donated). Violin plots represent kernel probability density of the data at different values (randomly jittered across the x-axis). Within each age group, the black squares represent the fixed effect estimates of first donations from the trial-level LMM, and error bars show the corresponding 95% confidence intervals. *** *p*_Bonf._ < 0.001.

*Section 8. Supplemental information 8: age trends in conforming probability are not entirely explained by response variance*

To control for potential age differences in response variance in the absence of social influence, one of our previous studies on risk perception (Knoll, Magis-Weinberg, Speekenbrink, & Blakemore, 2015) included a condition in which, after providing baseline ratings, participants observed ratings that were identical to their own and were subsequently asked to provide ratings once again. There were no age differences in this control condition: participants occasionally changed their ratings even in the absence of social influence, however there were no age differences in the extent to which this occurred. In addition, the study showed that, when the ratings of others differed from one’s own (thus in the presence of social influence), conformity to the risk perceptions of others decreased with age over and above any inter-individual differences in response variance. Having controlled for this, and to reduce task duration, a second study omitted this control condition (Knoll, Leung, Foulkes, & Blakemore, 2017). We reasoned similarly for the current study and therefore, by design, the observed donations of this study were never identical to participants’ first donations.

However, to obtain an alternative measure of response variability under social influence (rather than under its absence as in the study described above), we coded responses as 1 if participants conformed, as 0 if they did not change, and as -1 if they anti-conformed. We then took the participant-level variance of this vector as a measure of response variability. Linear regression showed that response variance was significantly associated with (the inverse of) age, suggesting that older participants were less likely to change their choices between phase 1 and phase 2 (slope = 2.87, SE = 0.83, p < 0.001).

Given that response variance was associated with age, we ran two (logistic mixed) models to inspect whether there were age trends not only in conforming probability (as reported in the manuscript) but also anti-conforming probability. Both models showed a significant positive relation between the inverse of age and the respective dependent variables (conforming: *χ*^2^(1) = 20.36, *p* < 0.001, slope = 18.34, SE = 4.06; anti-conforming: *χ*^2^(1) = 6.50, *p* = 0.011, slope = 12.61, SE = 4.95). These analyses suggest that age decreases conforming *and* anti-conforming probability.

However, we then ran two further control models to inspect whether these effects are robust when controlling for response variance. We found that controlling for response variance entirely explained the effect of age on anti-conforming probability (*χ*^2^(1) = 0.22, *p* = 0.637, slope = -1.20, SE = 2.54), but not conforming probability (*χ*^2^(1) = 6.76, *p* = 0.009, slope = 8.95, SE = 3.44).

Overall, these analyses suggest that age is associated with decreased response variance between adolescence and early adulthood, and that this contributes to but does not entirely explain decreasing conforming probability in this age range. We additionally ran a control model for each dependent variable suggesting that all reported age effects are robust to controlling for inter-individual differences in response variance (see CM11 in Section 5 and Tables S12-S17).

*Section 9. Supplemental information 9: opportunistic conformity in adults*

All our dependent variables showed effects that are consistent with opportunistic conformity in adults: adults were more influenced (in both influence magnitude and influence probability) when the observed donation from others was lower than their initial donation than when the observed donation was higher. This should be interpreted with caution given that it might have been modulated by the relatively low baseline donations of adults in our sample. These baseline donations were, by design, outside our control and, though our adaptive algorithm attempted to balance the number of prosocial and selfish deltas, there was a residual imbalance. In particular, such lower baseline donations led prosocial deltas to be larger than selfish deltas in the adult age group. This imbalance would predict that adults would show more prosocial influence than selfish influence, while we observed the opposite. Another possibility is that some prosocial deltas might have been judged as too extreme by adults to elicit a change in behaviour (Shang & Croson, 2009). However, if this were the case, we would expect to have observed a non-linear (for example, quadratic) relation between prosocial deltas and social influence, but our data showed a linear relationship.

**Supplemental Tables**

*Table S1. Main effect of age group on donations in phase 1: contrasts*

| **Contrast** | **Estimate** | **SE** | $\boldsymbol{P}_{\boldsymbol{uncorr}}$ | $\boldsymbol{P}_{\boldsymbol{Bonf}}$ |
| --- | --- | --- | --- | --- |
| YA - MA | -1.54 | 1.88 | 0.414 | 1 |
| YA - Ad | 13.19 | 1.98 | < 0.001 | < 0.001 |
| MA - Ad | 14.73 | 1.85 | < 0.001 | < 0.001 |

*Note.* YA = Young adolescents; MA = Mid adolescents; Ad = Adults

*Table S2. Main effect of source on influence probability (age categorical): contrasts*

| **Contrast** | **Estimate** | **SE** | $\boldsymbol{P}_{\boldsymbol{uncorr}}$ | $\boldsymbol{P}_{\boldsymbol{Bonf}}$ |
| --- | --- | --- | --- | --- |
| Teenagers - Adults | 0.05 | 0.07 | 0.515 | 1 |
| Teenagers - Computer | 0.48 | 0.08 | < 0.001 | < 0.001 |
| Adults - Computer | 0.43 | 0.08 | < 0.001 | < 0.001 |

*Table S3. Main effect of age group on influence probability (age categorical): contrasts*

| **Contrast** | **Estimate** | **SE** | $\boldsymbol{P}_{\boldsymbol{uncorr}}$ | $\boldsymbol{P}_{\boldsymbol{Bonf}}$ |
| --- | --- | --- | --- | --- |
| YA - MA | 0.63 | 0.18 | < 0.001 | 0.001 |
| YA - Ad | 0.67 | 0.19 | 0.001 | 0.002 |
| MA - Ad | 0.04 | 0.18 | 0.844 | 1 |

*Note.* YA = Young adolescents; MA = Mid adolescents; Ad = Adults

*Table S4a. Interaction between age group and direction of influence on influence probability (age categorical): between age group contrasts*

| **Contrast** | **Direction** | **Estimate** | **SE** | $\boldsymbol{P}_{\boldsymbol{uncorr}}$ | $\boldsymbol{P}_{\boldsymbol{Bonf}}$ |
| --- | --- | --- | --- | --- | --- |
| YA - MA | Prosocial | 0.65 | 0.25 | 0.01 | 0.062 |
| YA - Ad | Prosocial | 1.05 | 0.27 | < 0.001 | < 0.001 |
| MA - Ad | Prosocial | 0.41 | 0.25 | 0.104 | 0.625 |
| YA - MA | Selfish | 0.62 | 0.26 | 0.018 | 0.11 |
| YA - Ad | Selfish | 0.28 | 0.29 | 0.328 | 1 |
| MA - Ad | Selfish | -0.34 | 0.27 | 0.212 | 1 |

*Note.* YA = Young adolescents; MA = Mid adolescents; Ad = Adults

*Table S4b. Interaction between age group and direction of influence on influence probability (age categorical): within age group contrasts*

| **Contrast** | **Age Group** | **Estimate** | **SE** | $\boldsymbol{P}_{\boldsymbol{uncorr}}$ | $\boldsymbol{P}_{\boldsymbol{Bonf}}$ |
| --- | --- | --- | --- | --- | --- |
| Prosocial - Selfish | YA | 0.02 | 0.28 | 0.935 | 1 |
| Prosocial - Selfish | MA | 0 | 0.24 | 0.985 | 1 |
| Prosocial - Selfish | Ad | -0.75 | 0.28 | 0.008 | 0.025 |

*Note.* YA = Young adolescents; MA = Mid adolescents; Ad = Adults

*Table S5a. Interaction between age group, direction and influence on response times (age categorical): between age group contrasts*

| **Contrast** | **Influenced or Not** | **Direction** | **Estimate** | **SE** | $\boldsymbol{P}_{\boldsymbol{uncorr}}$ | $\boldsymbol{P}_{\boldsymbol{Bonf}}$ |
| --- | --- | --- | --- | --- | --- | --- |
| YA - MA | Inf | Prosocial | -0.09 | 0.06 | 0.145 | 1 |
| YA - Ad | Inf | Prosocial | 0.04 | 0.07 | 0.596 | 1 |
| MA - Ad | Inf | Prosocial | 0.13 | 0.06 | 0.051 | 0.609 |
| YA - MA | Not inf | Prosocial | -0.05 | 0.05 | 0.336 | 1 |
| YA - Ad | Not inf | Prosocial | 0.17 | 0.05 | 0.001 | 0.013 |
| MA - Ad | Not inf | Prosocial | 0.22 | 0.05 | < 0.001 | < 0.001 |
| YA - MA | Inf | Selfish | -0.11 | 0.06 | 0.058 | 0.702 |
| YA - Ad | Inf | Selfish | 0.05 | 0.06 | 0.435 | 1 |
| MA - Ad | Inf | Selfish | 0.16 | 0.06 | 0.008 | 0.098 |
| YA - MA | Not inf | Selfish | 0.04 | 0.05 | 0.398 | 1 |
| YA - Ad | Not inf | Selfish | 0.03 | 0.06 | 0.628 | 1 |
| MA - Ad | Not inf | Selfish | -0.02 | 0.05 | 0.752 | 1 |

*Note.* YA = Young adolescents; MA = Mid adolescents; Ad = Adults; Inf = Influenced; Not inf = Not influenced

*Table S5b. Interaction between age group, direction and influence on response times (age categorical): within age group contrasts*

| **Contrast** | **Influenced or Not** | **Age Group** | **Estimate** | **SE** | $\boldsymbol{P}_{\boldsymbol{uncorr}}$ | $\boldsymbol{P}_{\boldsymbol{Bonf}}$ |
| --- | --- | --- | --- | --- | --- | --- |
| Prosocial - Selfish | Inf | YA | -0.01 | 0.04 | 0.747 | 1 |
| Prosocial - Selfish | Not inf | YA | -0.03 | 0.04 | 0.370 | 1 |
| Prosocial - Selfish | Inf | MA | -0.03 | 0.04 | 0.399 | 1 |
| Prosocial - Selfish | Not inf | MA | 0.06 | 0.03 | 0.062 | 0.374 |
| Prosocial - Selfish | Inf | Ad | 0.00 | 0.05 | 0.990 | 1 |
| Prosocial - Selfish | Not inf | Ad | -0.18 | 0.04 | < 0.001 | < 0.001 |

*Note.* YA = Young adolescents; MA = Mid adolescents; Ad = Adults; Inf = Influenced; Not inf = Not influenced

*Table S6. Interaction between linear/quadratic age trends, direction and influence on response times (age continuous): contrasts*

| **Age Trend** | **Contrast** | **Direction** | **Estimate** | **SE** | $\boldsymbol{P}_{\boldsymbol{uncorr}}$ | $\boldsymbol{P}_{\boldsymbol{Bonf}}$ |
| --- | --- | --- | --- | --- | --- | --- |
| Linear | Inf - Not inf | Prosocial | 6.80 | 2.01 | 0.001 | 0.003 |
| Linear | Inf - Not inf | Selfish | -3.26 | 2.06 | 0.113 | 0.452 |
| quadratic | Inf - Not inf | Prosocial | 3.71 | 2.00 | 0.064 | 0.258 |
| quadratic | Inf - Not inf | Selfish | -5.21 | 1.96 | 0.008 | 0.031 |

*Note,* Inf = Influenced; Not inf = Not influenced

*Table S7. Main effect of source on influence magnitude (age categorical): contrasts*

| **Contrast** | **Estimate** | **SE** | $\boldsymbol{P}_{\boldsymbol{uncorr}}$ | $\boldsymbol{P}_{\boldsymbol{Bonf}}$ |
| --- | --- | --- | --- | --- |
| Teenagers - Adults | 0.22 | 0.16 | 0.172 | 0.515 |
| Teenagers - Computer | 0.86 | 0.19 | < 0.001 | < 0.001 |
| Adults - Computer | 0.64 | 0.15 | < 0.001 | < 0.001 |

*Table S8. Interaction between age group and direction of influence on slopes of influence magnitude (age categorical): between age group contrasts*

| **Contrast** | **Direction** | **Estimate** | **SE** | $\boldsymbol{P}_{\boldsymbol{uncorr}}$ | $\boldsymbol{P}_{\boldsymbol{Bonf}}$ |
| --- | --- | --- | --- | --- | --- |
| YA - MA | Prosocial | 0.00 | 0.03 | 0.907 | 1 |
| YA - Ad | Prosocial | 0.03 | 0.03 | 0.220 | 1 |
| MA - Ad | Prosocial | 0.03 | 0.02 | 0.224 | 1 |
| YA - MA | Selfish | 0.07 | 0.03 | 0.007 | 0.042 |
| YA - Ad | Selfish | -0.03 | 0.03 | 0.389 | 1 |
| MA - Ad | Selfish | -0.10 | 0.03 | 0.001 | 0.008 |

*Note.* YA = Young adolescents; MA = Mid adolescents; Ad = Adults

*Table S9. Main effect of source on influence magnitude (age continuous): contrasts*

| **Contrast** | **Estimate** | **SE** | $\boldsymbol{P}_{\boldsymbol{uncorr}}$ | $\boldsymbol{P}_{\boldsymbol{Bonf}}$ |
| --- | --- | --- | --- | --- |
| Teenagers - Adults | 0.26 | 0.16 | 0.110 | 0.331 |
| Teenagers - Computer | 0.90 | 0.19 | < 0.001 | < 0.001 |
| Adults - Computer | 0.64 | 0.15 | < 0.001 | < 0.001 |

*Table S10 First Donations (Age Categorical)*

|  | **RfM** | **RfM + Gender** | **RfM + Conversion Rate Guess** | **RfM + Abstract Reasoning** | **RfM + Group Size** |
| --- | --- | --- | --- | --- | --- |
| *Intercept* | 24.73***  (1.42) | 28.43***  (1.91) | 24.73***  (1.42) | 25.31***  (1.44) | 26.74***  (1.53) |
| *Age Group:* Mid Adolescents | 1.54  (1.88) | -0.21  (2.62) | 1.54  (1.89) | 0.36  (1.96) | 1.72  (1.84) |
| *Age Group:* Adults | -13.19***  (1.98) | -13.96***  (2.73) | -13.19***  (1.99) | -13.58***  (1.98) | -19.82***  (2.85) |
| *Gender:* Male |  | -7.78**  (2.77) |  |  |  |
| *Age Group:* Mid Adolescents x *Gender:* Male |  | 4.19  (3.68) |  |  |  |
| *Age Group:* Adults x *Gender:* Male |  | 2.15  (3.86) |  |  |  |
| *Conversion Rate Guess* |  |  | 0  (0.78) |  |  |
| *MaRs-IB* |  |  |  | 1.62*  (0.81) |  |
| *Group Size* |  |  |  |  | -3.94**  (1.24) |

*Note.* *** p<0.001; ** p<0.01; * p<0.05; · p<0.1; (SE).

*Table S11. First Donations (Age Continuous)*

|  | **RfM** | **RfM + Gender** | **RfM + Conversion Rate Guess** | **RfM + Abstract Reasoning** | **RfM + Group Size** |
| --- | --- | --- | --- | --- | --- |
| *Intercept* | 21.18***  (0.78) | 24.33***  (1.1) | 21.41***  (0.81) | 21.18***  (0.78) | 21.19***  (0.78) |
| *Age Trend:* Linear | -519.44***  (69.51) | -448.71***  (103.76) | -518.64***  (69.72) | -518.34***  (69) | -676.05***  (102.98) |
| *Age Trend:* Quadratic | -45.81  (69.52) | 105  (117.81) | -47.24  (69.84) | -3.17  (72.02) | -32.62  (69.31) |
| *Age Trend:* Cubic | 216.15**  (69.52) | 332.43**  (117.81) | 214.02**  (70.06) | 186.49**  (70.48) | 270.68***  (73.97) |
| *Gender:* Male |  | -5.91***  (1.53) |  |  |  |
| *Age Trend:* Linear x *Gender:* Male |  | -94.75  (139.81) |  |  |  |
| *Age Trend:* Quadratic x *Gender:* Male |  | -197.46  (146.61) |  |  |  |
| *Age Trend:* Cubic x *Gender:* Male |  | -111.34  (147.7) |  |  |  |
| *Conversion Rate Guess* |  |  | 0.23  (0.79) |  |  |
| *MaRs-IB* |  |  |  | 1.71*  (0.83) |  |
| *Group Size* |  |  |  |  | -2.45*  (1.2) |

*Note.* *** p<0.001; ** p<0.01; * p<0.05; · p<0.1; (SE).

*Table S12. Influence Probability (Age Categorical)*

|  | **RfM** | **RfM + Gender** | **RfM + Guess conversion rate** | **RfM + Abstract reasoning** | **RfM+ First donation** | **RfM + Δ** | **RfM + Converted Δ** | **RfM + Block order** | **RfM + Group size** | **RfM + Response variance** | **RfM^1^ + Non-social influence** |
| --- | --- | --- | --- | --- | --- | --- | --- | --- | --- | --- | --- |
| *Intercept* | -0.26  (0.2) | -0.32  (0.23) | -0.26  (0.19) | -0.3  (0.2) | -0.28  (0.2) | -0.24  (0.2) | -0.24  (0.2) | -0.05  (0.21) | -0.27  (0.21) | -1.6***  (0.23) | -0.51**  (0.18) |
| *Age Group:*  Mid Adolescents | -0.65*  (0.25) | -0.63*  (0.31) | -0.67**  (0.25) | -0.57*  (0.26) | -0.65*  (0.25) | -0.69**  (0.26) | -0.7**  (0.25) | -0.64*  (0.25) | -0.65*  (0.25) | -0.36  (0.24) | -0.26  (0.23) |
| *Age Group:* Adults | -1.05***  (0.27) | -0.89**  (0.33) | -1.03***  (0.26) | -1.03***  (0.27) | -1.1***  (0.27) | -1.19***  (0.27) | -1.09***  (0.27) | -1.05***  (0.27) | -1.03**  (0.34) | -0.72**  (0.25) | -0.69**  (0.24) |
| *Source:* Adults | -0.05  (0.07) | -0.05  (0.07) | -0.05  (0.07) | -0.05  (0.07) | -0.05  (0.07) | -0.05  (0.08) | -0.05  (0.07) | -0.05  (0.07) | -0.05  (0.07) | -0.05  (0.07) | -0.05  (0.07) |
| *Source:* Computer | -0.48***  (0.08) | -0.48***  (0.08) | -0.48***  (0.08) | -0.48***  (0.08) | -0.48***  (0.08) | -0.49***  (0.08) | -0.49***  (0.08) | -0.48***  (0.08) | -0.48***  (0.08) | -0.49***  (0.08) |  |
| *Direction:* Selfish | -0.02  (0.28) | -0.02  (0.28) | -0.02  (0.28) | -0.02  (0.28) | 0.04  (0.28) | -0.04  (0.29) | -0.04  (0.28) | -0.02  (0.28) | -0.02  (0.28) | -0.02  (0.27) | -0.03  (0.27) |
| *Age Group:* Mid Adolescents x *Direction:* Selfish | 0.03  (0.37) | 0.03  (0.37) | 0.02  (0.37) | 0.03  (0.37) | 0.05  (0.37) | 0.02  (0.38) | 0.04  (0.38) | 0.02  (0.37) | 0.03  (0.37) | 0.04  (0.36) | 0.06  (0.36) |
| *Age Group:* Adults x *Direction:* Selfish | 0.77·  (0.4) | 0.77·  (0.4) | 0.77·  (0.4) | 0.78*  (0.4) | 0.77·  (0.4) | 1.06*  (0.41) | 0.91*  (0.41) | 0.77·  (0.4) | 0.77·  (0.4) | 0.67·  (0.38) | 0.61  (0.38) |
| *Gender:* Male |  | 0.12  (0.27) |  |  |  |  |  |  |  |  |  |
| *Age Group:* Mid Adolescents x *Gender:* Male |  | -0.04  (0.36) |  |  |  |  |  |  |  |  |  |
| *Age Group:* Adults x *Gender:* Male |  | -0.32  (0.38) |  |  |  |  |  |  |  |  |  |
| *Guess Conversion Rate* |  |  | 0.17*  (0.07) |  |  |  |  |  |  |  |  |
| *Mars-IB* |  |  |  | -0.1  (0.08) |  |  |  |  |  |  |  |
| *First Donation* |  |  |  |  | -0.07  (0.06) |  |  |  |  |  |  |
| *Δ* |  |  |  |  |  | 0.3***  (0.03) |  |  |  |  |  |
| *Converted Δ* |  |  |  |  |  |  | 0.3***  (0.04) |  |  |  |  |
| *Block* |  |  |  |  |  |  |  | -0.11**  (0.04) |  |  |  |
| *Group Size* |  |  |  |  |  |  |  |  | 0.01  (0.12) |  |  |
| *Variance in Conformity* |  |  |  |  |  |  |  |  |  | 3.18***  (0.34) |  |
| *Computer Influence* |  |  |  |  |  |  |  |  |  |  | 0.76***  (0.06) |

*Note.* *** p<0.001; ** p<0.01; * p<0.05; · p<0.1; (SE). ^1^This model focuses on social influence trials only.

*Table S13 Influence Probability (Age Continuous)*

|  | **RfM** | **RfM + Gender** | **RfM + Guess conversion rate** | **RfM + Abstract reasoning** | **RfM+ First donation** | **RfM + Δ** | **RfM + Converted Δ** | **RfM + Block order** | **RfM + Group size** | **RfM + Response variance** | **RfM^1^+ Non-social influence** |
| --- | --- | --- | --- | --- | --- | --- | --- | --- | --- | --- | --- |
| *Intercept* | -2.21***  (0.33) | -2***  (0.43) | -2.18***  (0.33) | -2.16***  (0.32) | -2.34***  (0.36) | -2.43***  (0.37) | -2.28***  (0.34) | -1.99***  (0.33) | -2.48***  (0.35) | -2.82***  (0.31) | -1.66***  (0.32) |
| *Age Trend:* Inverse | 23.21***  (5.15) | 20.03**  (6.97) | 22.73***  (5.21) | 22.33***  (5.07) | 24.7***  (5.65) | 26.46***  (5.87) | 24.2***  (5.42) | 23.08***  (5.04) | 27.89***  (5.7) | 14.04**  (4.72) | 14.28*  (5.13) |
| *Direction:* Selfish | 1.03*  (0.44) | 1.03*  (0.46) | 1.03*  (0.47) | 1.05*  (0.42) | 1.12*  (0.48) | 1.5**  (0.54) | 1.26*  (0.5) | 1.03*  (0.43) | 1.04*  (0.45) | 0.88*  (0.41) | 0.78  (0.63) |
| *Source:* Adults | -0.05  (0.07) | -0.05  (0.07) | -0.05  (0.07) | -0.05  (0.07) | -0.05  (0.07) | -0.05  (0.08) | -0.05  (0.07) | -0.05  (0.07) | -0.05  (0.07) | -0.05  (0.07) | -0.05  (0.07) |
| *Source:* Computer | -0.48***  (0.08) | -0.48***  (0.08) | -0.49***  (0.08) | -0.48***  (0.08) | -0.48***  (0.08) | -0.49***  (0.08) | -0.49***  (0.08) | -0.48***  (0.08) | -0.49***  (0.08) | -0.49***  (0.08) |  |
| *Age Trend:* Inverse x *Direction:* Selfish | -13.87*  (6.98) | -13.79·  (7.38) | -13.87·  (7.49) | -14.06*  (6.61) | -13.85·  (7.56) | -20.58*  (8.59) | -17.22*  (7.99) | -13.8*  (6.71) | -14.03*  (7.04) | -11.53·  (6.42) | -10.28  (10.43) |
| *Gender:* Male |  | -0.41  (0.57) |  |  |  |  |  |  |  |  |  |
| *Age Trend:* Inverse x *Gender:* Male |  | 6.25  (9.2) |  |  |  |  |  |  |  |  |  |
| *Guess Conversion Rate* |  |  | 0.15*  (0.08) |  |  |  |  |  |  |  |  |
| *Mars-IB* |  |  |  | -0.14·  (0.08) |  |  |  |  |  |  |  |
| *First Donation* |  |  |  |  | -0.09  (0.06) |  |  |  |  |  |  |
| *Δ* |  |  |  |  |  | 0.29***  (0.03) |  |  |  |  |  |
| *Converted Δ* |  |  |  |  |  |  | 0.29***  (0.04) |  |  |  |  |
| *Block* |  |  |  |  |  |  |  | -0.11**  (0.04) |  |  |  |
| *Group Size* |  |  |  |  |  |  |  |  | -0.11  (0.09) |  |  |
| *Variance in Conformity* |  |  |  |  |  |  |  |  |  | 3.25***  (0.34) |  |
| *Computer Influence* |  |  |  |  |  |  |  |  |  |  | 0.76***  (0.06) |

*Note.* *** p<0.001; ** p<0.01; * p<0.05; · p<0.1; (SE). ^1^This model focuses on social influence trials only.

*Table S14 Response Times (Age Categorical)*

|  | **RfM** | **RfM + Gender** | **RfM + Guess conversion rate** | **RfM + Abstract reasoning** | **RfM+ First donation** | **RfM + Δ** | **RfM + Converted Δ** | **RfM + Block order** | **RfM + Group size** | **RfM + Response variance** | **RfM + RT during first donation** |
| --- | --- | --- | --- | --- | --- | --- | --- | --- | --- | --- | --- |
| *Intercept* | 8.09***  (0.05) | 8.18***  (0.06) | 8.09***  (0.05) | 8.09***  (0.05) | 8.09***  (0.05) | 8.1***  (0.05) | 8.09*** (0.05) | 8.17***  (0.05) | 8.16***  (0.05) | 8.08*** (0.06) | 6.66***  (0.09) |
| *Age Group:* Mid Adolescents | 0.09  (0.06) | 0.1  (0.08) | 0.09  (0.06) | 0.09  (0.07) | 0.09  (0.06) | 0.09  (0.06) | 0.09  (0.06) | 0.09  (0.06) | 0.1  (0.06) | 0.09  (0.06) | 0.08  (0.06) |
| *Age Group:* Adults | -0.04  (0.07) | -0.06  (0.08) | -0.04  (0.07) | -0.03  (0.07) | -0.05  (0.07) | -0.03  (0.07) | -0.04  (0.07) | -0.03  (0.07) | -0.25**  (0.08) | -0.03  (0.07) | 0.01  (0.06) |
| *Contagion:* Not influenced | -0.11**  (0.04) | -0.1**  (0.04) | -0.11**  (0.04) | -0.1**  (0.04) | -0.11**  (0.04) | -0.11**  (0.04) | -0.11**  (0.04) | -0.1**  (0.04) | -0.11**  (0.04) | -0.1**  (0.04) | -0.1**  (0.04) |
| *Direction:* Selfish | 0.01  (0.04) | 0.01  (0.04) | 0.01  (0.04) | 0.01  (0.04) | 0.03  (0.04) | 0.01  (0.04) | 0.01  (0.04) | 0.02  (0.04) | 0.01  (0.04) | 0.01  (0.04) | 0.02  (0.04) |
| *Source:* Adults | 0.02·  (0.01) | 0.02·  (0.01) | 0.02·  (0.01) | 0.02·  (0.01) | 0.02·  (0.01) | 0.02·  (0.01) | 0.02·  (0.01) | 0.02·  (0.01) | 0.02·  (0.01) | 0.02·  (0.01) | 0.02  (0.01) |
| *Source:* Computer | -0.01  (0.02) | -0.01  (0.01) | -0.01  (0.02) | -0.01  (0.01) | -0.01  (0.02) | -0.01  (0.02) | -0.01  (0.02) | -0.01  (0.02) | -0.01  (0.02) | -0.01  (0.01) | -0.01  (0.02) |
| *Age Group:* Mid Adolescents x *Contagion:* Not influenced | -0.04  (0.05) | -0.05  (0.05) | -0.04  (0.05) | -0.05  (0.05) | -0.04  (0.05) | -0.04  (0.05) | -0.04  (0.05) | -0.04  (0.05) | -0.04  (0.05) | -0.05  (0.05) | -0.05  (0.05) |
| *Age Group:* Adults x *Contagion:* Not influenced | -0.13*  (0.06) | -0.15*  (0.06) | -0.13*  (0.06) | -0.15**  (0.06) | -0.13*  (0.06) | -0.13*  (0.06) | -0.13*  (0.06) | -0.13*  (0.06) | -0.13*  (0.06) | -0.15**  (0.06) | -0.12*  (0.05) |
| *Age Group:* Mid Adolescents x *Direction:* Selfish | 0.02  (0.06) | 0.02  (0.06) | 0.02  (0.06) | 0.03  (0.06) | 0.03  (0.06) | 0.02  (0.06) | 0.02  (0.06) | 0.02  (0.06) | 0.02  (0.06) | 0.03  (0.06) | 0.04  (0.05) |
| *Age Group:* Adults x *Direction:* Selfish | -0.01  (0.06) | -0.02  (0.07) | -0.01  (0.06) | -0.02  (0.07) | -0.01  (0.06) | -0.02  (0.07) | -0.02  (0.07) | -0.02  (0.06) | -0.01  (0.06) | -0.02  (0.07) | -0.04  (0.06) |
| *Contagion:* Not influenced x *Direction:* Selfish | 0.02  (0.05) | 0.01  (0.05) | 0.02  (0.05) | 0.01  (0.05) | 0.02  (0.05) | 0.02  (0.05) | 0.02  (0.05) | 0.02  (0.05) | 0.02  (0.05) | 0.01  (0.05) | 0.02  (0.05) |
| *Age Group:* Mid Adolescents x *Direction:* Selfish x *Contagion:* Not influenced | -0.11·  (0.06) | -0.1  (0.06) | -0.11·  (0.06) | -0.1  (0.06) | -0.11·  (0.06) | -0.11·  (0.06) | -0.11·  (0.06) | -0.12·  (0.06) | -0.12·  (0.06) | -0.1  (0.06) | -0.12·  (0.06) |
| *Age Group:* Adults x *Direction:* Selfish x *Contagion:* Not influenced | 0.16*  (0.07) | 0.17*  (0.07) | 0.16*  (0.07) | 0.17*  (0.07) | 0.15*  (0.07) | 0.16*  (0.07) | 0.16*  (0.07) | 0.16*  (0.07) | 0.16*  (0.07) | 0.17*  (0.07) | 0.14*  (0.07) |
| *Gender:* Male |  | -0.17**  (0.07) |  |  |  |  |  |  |  |  |  |
| *Age Group:* Mid Adolescents x *Gender*: Male |  | 0  (0.09) |  |  |  |  |  |  |  |  |  |
| *Age Group:* Adults x *Gender:* Male |  | 0.06  (0.09) |  |  |  |  |  |  |  |  |  |
| *Conversion Rate Guess* |  |  | 0  (0.02) |  |  |  |  |  |  |  |  |
| *Mars-IB* |  |  |  | 0  (0.02) |  |  |  |  |  |  |  |
| *First Donation* |  |  |  |  | -0.03*  (0.01) |  |  |  |  |  |  |
| *Δ* |  |  |  |  |  | 0  (0.01) |  |  |  |  |  |
| *Converted Δ* |  |  |  |  |  |  | 0  (0.01) |  |  |  |  |
| *Block* |  |  |  |  |  |  |  | -0.04***  (0.01) |  |  |  |
| *Group Size* |  |  |  |  |  |  |  |  | -0.13***  (0.03) |  |  |
| *Variance in Conformity* |  |  |  |  |  |  |  |  |  | 0.04  (0.09) |  |
| *Response Time at First Donation* |  |  |  |  |  |  |  |  |  |  | 0.17***  (0.01) |

*Note.* *** p<0.001; ** p<0.01; * p<0.05; · p<0.1; (SE).

*Table S15 Response Times (Age Continuous)*

|  | **RfM** | **RfM + Gender** | **RfM + Guess conversion rate** | **RfM + Abstract reasoning** | **RfM+ First donation** | **RfM + Δ** | **RfM + Converted Δ** | **RfM + Block order** | **RfM + Group size** | **RfM + Response variance** | **RfM + RT during first donation** |
| --- | --- | --- | --- | --- | --- | --- | --- | --- | --- | --- | --- |
| *Intercept* | 8.12***  (0.03) | 8.19***  (0.03) | 8.12***  (0.03) | 8.12***  (0.03) | 8.11***  (0.03) | 8.12***  (0.03) | 8.12***  (0.03) | 8.19***  (0.03) | 8.12***  (0.03) | 8.1***  (0.04) | 6.69***  (0.08) |
| *Age Trend:* Linear | -0.54  (2.4) | -2.27  (2.92) | -0.53  (2.4) | -0.54  (2.4) | -1.23  (2.44) | -0.48  (2.4) | -0.54  (2.4) | -0.22  (2.46) | -5.39·  (2.91) | -0.37  (2.43) | 1.03  (2.16) |
| *Age Trend:* Quadratic | 0.6  (2.38) | -3.18  (3.1) | 0.59  (2.38) | 0.63  (2.43) | 0.62  (2.41) | 0.59  (2.38) | 0.6  (2.38) | 0.64  (2.43) | 1.07  (2.39) | 0.53  (2.39) | 0.4  (2.13) |
| *Contagion:* Not influenced | -0.17***  (0.02) | -0.17***  (0.02) | -0.17***  (0.02) | -0.17***  (0.02) | -0.17***  (0.02) | -0.17***  (0.02) | -0.17***  (0.02) | -0.16***  (0.02) | -0.17***  (0.02) | -0.17***  (0.02) | -0.16***  (0.02) |
| *Direction:* Selfish | 0.02  (0.03) | 0.02  (0.03) | 0.02  (0.03) | 0.02  (0.03) | 0.04  (0.03) | 0.01  (0.03) | 0.02  (0.03) | 0.02  (0.03) | 0.02  (0.03) | 0.02  (0.03) | 0.02  (0.02) |
| *Source:* Adults | 0.02·  (0.01) | 0.02·  (0.01) | 0.02·  (0.01) | 0.02·  (0.01) | 0.02·  (0.01) | 0.02·  (0.01) | 0.02·  (0.01) | 0.02·  (0.01) | 0.02·  (0.01) | 0.02·  (0.01) | 0.02  (0.01) |
| *Source:* Computer | -0.01  (0.02) | -0.01  (0.02) | -0.01  (0.02) | -0.01  (0.02) | -0.01  (0.02) | -0.01  (0.02) | -0.01  (0.02) | -0.01  (0.01) | -0.01  (0.02) | -0.01  (0.02) | -0.01  (0.02) |
| *Age Trend:* Linear x *Contagion:* Not influenced | -6.8***  (2.01) | -6.74***  (2.01) | -6.8***  (2.01) | -6.8***  (2.01) | -6.79***  (2.01) | -6.79***  (2.01) | -6.8***  (2.01) | -7.33***  (2.04) | -6.86***  (2.01) | -6.78***  (2.01) | -5.93**  (1.93) |
| *Age Trend:* Quadratic x *Contagion:* Not influenced | -3.71·  (2) | -3.76·  (2) | -3.71·  (2) | -3.71·  (2) | -3.7·  (2.01) | -3.71·  (2) | -3.71·  (2) | -3.35  (2.03) | -3.75·  (2) | -3.71·  (2) | -2.57  (1.93) |
| *Age Trend:* Linear x *Direction:* Selfish | -3.37  (2.37) | -3.38  (2.37) | -3.37  (2.37) | -3.38  (2.37) | -3.38  (2.36) | -3.51  (2.37) | -3.37  (2.37) | -3.75  (2.39) | -3.39  (2.37) | -3.38  (2.37) | -4.72*  (2.2) |
| *Age Trend:* Quadratic x *Direction:* Selfish | -5.5*  (2.26) | -5.48*  (2.26) | -5.5*  (2.26) | -5.5*  (2.26) | -5.6*  (2.26) | -5.49*  (2.26) | -5.5*  (2.26) | -5.6*  (2.27) | -5.6*  (2.26) | -5.49*  (2.26) | -5.89**  (2.09) |
| *Contagion:* Not influenced x *Direction:* Selfish | 0.02  (0.03) | 0.02  (0.03) | 0.02  (0.03) | 0.02  (0.03) | 0.02  (0.03) | 0.02  (0.03) | 0.02  (0.03) | 0.02  (0.03) | 0.02  (0.03) | 0.02  (0.03) | 0.02  (0.03) |
| *Age Trend:* Linear x *Direction:* Selfish x *Contagion:* Not influenced | 10.06***  (2.49) | 10***  (2.49) | 10.06***  (2.49) | 10.06***  (2.49) | 9.88***  (2.49) | 10.03***  (2.49) | 10.06***  (2.49) | 10.76***  (2.5) | 10.16***  (2.49) | 10.03***  (2.49) | 9.75***  (2.42) |
| *Age Trend:* Quadratic x *Direction:* Selfish x *Contagion:* Not influenced | 8.92***  (2.4) | 8.93***  (2.4) | 8.92***  (2.4) | 8.92***  (2.4) | 8.76***  (2.4) | 8.89***  (2.4) | 8.92***  (2.4) | 8.4***  (2.41) | 8.91***  (2.4) | 8.91***  (2.4) | 8.62***  (2.33) |
| *Gender:* Male |  | -0.14***  (0.03) |  |  |  |  |  |  |  |  |  |
| *Age Group:* Mid Adolescents x *Gender:* Male |  | 2.49  (3.12) |  |  |  |  |  |  |  |  |  |
| *Age Group:* Adults x *Gender:* Male |  | 6.32·  (3.21) |  |  |  |  |  |  |  |  |  |
| *Conversion Rate Guess* |  |  | 0  (0.02) |  |  |  |  |  |  |  |  |
| *Mars-IB* |  |  |  | 0  (0.02) |  |  |  |  |  |  |  |
| *First Donation* |  |  |  |  | -0.03*  (0.01) |  |  |  |  |  |  |
| *Δ* |  |  |  |  |  | 0  (0.01) |  |  |  |  |  |
| *Converted Δ* |  |  |  |  |  |  | 0  (0.01) |  |  |  |  |
| *Block* |  |  |  |  |  |  |  | -0.04***  (0.01) |  |  |  |
| *Group Size* |  |  |  |  |  |  |  |  | -0.08**  (0.03) |  |  |
| *Variance in Conformity* |  |  |  |  |  |  |  |  |  | 0.04  (0.09) |  |
| *Response Time at First Donation* |  |  |  |  |  |  |  |  |  |  | 0.17***  (0.01) |

*Note.* *** p<0.001; ** p<0.01; * p<0.05; · p<0.1; (SE).

*Table S16. Influence Magnitude (Age Categorical)*

|  | **RfM** | **RfM + Gender** | **RfM + Guess conversion rate** | **RfM + Abstract reasoning** | **RfM+ First donation** | **RfM + Converted Δ** | **RfM + Block order** | **RfM + Group size** | **RfM + Response variance** | **RfM^1^+ Non-social influence** | **RfM^2^** |
| --- | --- | --- | --- | --- | --- | --- | --- | --- | --- | --- | --- |
| *Intercept* | 2.48***  (0.48) | 2.23***  (0.53) | 2.49***  (0.47) | 2.38***  (0.48) | 2.54***  (0.48) | 0.11  (0.07) | 2.98***  (0.5) | 2.47***  (0.49) | 3.06***  (0.55) | 2.47***  (0.46) | 2.05***  (0.31) |
| *Age Group:* Mid Adolescents | -0.71  (0.63) | -0.33  (0.71) | -0.75  (0.63) | -0.53  (0.64) | -0.71  (0.63) | -0.12  (0.06) | -0.71  (0.63) | -0.71  (0.63) | -0.84  (0.64) | -0.56  (0.61) | -0.5  (0.42) |
| *Age Group:* Adults | -0.29  (0.66) | -0.34  (0.73) | -0.25  (0.65) | -0.22  (0.65) | -0.16  (0.66) | 0.05  (0.1) | -0.26  (0.66) | -0.25  (0.74) | -0.44  (0.66) | -0.35  (0.63) | -0.57  (0.43) |
| *Direction*: Selfish | 0.6  (0.66) | 0.59  (0.66) | 0.6  (0.66) | 0.6  (0.66) | 0.39  (0.66) | -0.02  (0.09) | 0.6  (0.66) | 0.6  (0.66) | 0.58  (0.66) | 0.47  (0.67) | 0.53  (0.5) |
| *Source:* Adults | -0.27  (0.38) | -0.28  (0.38) | -0.27  (0.38) | -0.27  (0.38) | -0.28  (0.38) | -0.03  (0.06) | -0.27  (0.38) | -0.27  (0.38) | -0.28  (0.38) | -0.29  (0.39) | -0.17  (0.29) |
| *Source:* Computer | -1*  (0.42) | -1*  (0.42) | -1*  (0.42) | -1.01*  (0.42) | -1.01*  (0.42) | -0.11·  (0.06) | -1*  (0.42) | -1*  (0.42) | -1*  (0.42) |  | -0.96**  (0.29) |
| *Δ* | 0.91***  (0.23) | 0.9***  (0.23) | 0.91***  (0.23) | 0.9***  (0.23) | 0.97***  (0.23) | 0.39***  (0.06) | 0.9***  (0.23) | 0.91***  (0.23) | 0.91***  (0.23) | 1.16***  (0.27) | 1.19***  (0.22) |
| *Age Group:* Mid Adolescents x *Direction:* Selfish | -0.24  (0.87) | -0.24  (0.87) | -0.26  (0.87) | -0.24  (0.87) | -0.28  (0.87) | 0.04  (0.12) | -0.25  (0.87) | -0.24  (0.87) | -0.23  (0.87) | -0.18  (0.89) | -0.38  (0.67) |
| *Age Group:* Adults x *Direction:* Selfish | -0.01  (0.94) | 0.02  (0.94) | -0.01  (0.94) | 0  (0.94) | -0.03  (0.94) | 0.04  (0.13) | -0.03  (0.94) | -0.01  (0.94) | 0.05  (0.94) | -0.23  (0.96) | -0.07  (0.72) |
| *Age Group:* Mid Adolescents x *Source:* Adults | -0.12  (0.51) | -0.12  (0.51) | -0.12  (0.51) | -0.12  (0.51) | -0.11  (0.51) | -0.01  (0.08) | -0.13  (0.5) | -0.12  (0.51) | -0.12  (0.51) | -0.11  (0.52) | -0.21  (0.39) |
| *Age Group*: Adults x *Source:* Adults | -0.33  (0.52) | -0.33  (0.52) | -0.33  (0.52) | -0.33  (0.52) | -0.32  (0.52) | 0.15·  (0.08) | -0.36  (0.51) | -0.33  (0.52) | -0.33  (0.52) | -0.32  (0.52) | 0  (0.39) |
| *Age Group:* Mid Adolescents x *Source:* Computer | 0.1  (0.56) | 0.1  (0.56) | 0.1  (0.56) | 0.11  (0.56) | 0.11  (0.56) | 0.01  (0.08) | 0.11  (0.56) | 0.1  (0.56) | 0.1  (0.56) |  | 0.03  (0.39) |
| *Age Group:* Adults x *Source:* Computer | -0.19  (0.57) | -0.2  (0.57) | -0.2  (0.57) | -0.2  (0.57) | -0.18  (0.57) | 0.12  (0.08) | -0.23  (0.57) | -0.19  (0.57) | -0.21  (0.57) |  | 0.35  (0.39) |
| *Direction:* Selfish x *Source:* Adults | -0.27  (0.48) | -0.27  (0.48) | -0.28  (0.48) | -0.27  (0.48) | -0.26  (0.48) | -0.03  (0.08) | -0.27  (0.48) | -0.27  (0.48) | -0.27  (0.48) | -0.23  (0.5) | -0.27  (0.42) |
| *Direction:* Selfish x *Source:* Computer | -0.63  (0.48) | -0.63  (0.48) | -0.63  (0.48) | -0.62  (0.48) | -0.61  (0.48) | -0.09  (0.08) | -0.63  (0.48) | -0.63  (0.48) | -0.63  (0.48) |  | -0.64  (0.42) |
| *Direction:* Selfish x *Δ* | 0.61*  (0.26) | 0.62*  (0.26) | 0.61*  (0.26) | 0.62*  (0.26) | 0.48·  (0.27) | -0.15*  (0.06) | 0.62*  (0.26) | 0.61*  (0.26) | 0.61*  (0.26) | 0.54·  (0.32) | 0.59**  (0.23) |
| *Age Group:* Mid Adolescents x *Δ* | -0.04  (0.3) | -0.03  (0.3) | -0.04  (0.3) | -0.03  (0.3) | -0.04  (0.3) | -0.22**  (0.08) | -0.03  (0.3) | -0.04  (0.3) | -0.03  (0.3) | 0.06  (0.36) | -0.27  (0.29) |
| *Age Group:* Adults x *Δ* | -0.37  (0.3) | -0.36  (0.3) | -0.37  (0.3) | -0.36  (0.3) | -0.42  (0.3) | -0.22**  (0.09) | -0.36  (0.3) | -0.37  (0.3) | -0.37  (0.3) | -0.56  (0.36) | -0.36  (0.29) |
| *Age Group:* Mid Adolescents x *Direction:* Selfish x *Source:* Adults | 0.69  (0.63) | 0.68  (0.63) | 0.69  (0.63) | 0.69  (0.63) | 0.68  (0.63) | 0.09  (0.11) | 0.7  (0.63) | 0.69  (0.63) | 0.68  (0.63) | 0.62  (0.66) | 0.65  (0.55) |
| *Age Group:* Adults x *Direction:* Selfish x *Source:* Adults | 1.32·  (0.68) | 1.33*  (0.68) | 1.32·  (0.68) | 1.33*  (0.68) | 1.31·  (0.68) | 0.24*  (0.12) | 1.35*  (0.67) | 1.32·  (0.68) | 1.34*  (0.68) | 1.31·  (0.7) | 0.76  (0.58) |
| *Age Group:* Mid Adolescents x *Direction:* Selfish x *Source*: Computer | 0.88  (0.63) | 0.88  (0.63) | 0.88  (0.63) | 0.87  (0.63) | 0.86  (0.63) | 0.12  (0.11) | 0.88  (0.63) | 0.88  (0.63) | 0.89  (0.63) |  | 0.96·  (0.55) |
| *Age Group:* Adults x *Direction:* Selfish x *Source:* Computer | 2.04**  (0.68) | 2.05**  (0.68) | 2.04**  (0.68) | 2.05**  (0.68) | 2.01**  (0.68) | 0.38**  (0.12) | 2.06**  (0.68) | 2.04**  (0.68) | 2.08**  (0.68) |  | 1.39*  (0.58) |
| *Age Group:* Mid Adolescents x *Direction:* Selfish x *Δ* | -0.76*  (0.34) | -0.77*  (0.34) | -0.75*  (0.34) | -0.77*  (0.34) | -0.73*  (0.34) | 0.21**  (0.08) | -0.75*  (0.34) | -0.76*  (0.34) | -0.76*  (0.34) | -1.13**  (0.41) | -0.49·  (0.29) |
| *Age Group:* Adults x *Direction:* Selfish x *Δ* | 0.68·  (0.39) | 0.68·  (0.39) | 0.68·  (0.39) | 0.68·  (0.39) | 0.67·  (0.39) | 0.56***  (0.09) | 0.68·  (0.39) | 0.68·  (0.39) | 0.68·  (0.39) | 0.57  (0.48) | 0.42  (0.34) |
| *Gender:* Male |  | 0.52  (0.47) |  |  |  |  |  |  |  |  |  |
| *Age Group:* Mid Adolescents x *Gender:* Male |  | -0.75  (0.62) |  |  |  |  |  |  |  |  |  |
| *Age Group:* Adults x *Gender*: Male |  | 0.06  (0.65) |  |  |  |  |  |  |  |  |  |
| *Conversion Rate Guess* |  |  | 0.33*  (0.13) |  |  |  |  |  |  |  |  |
| *Mars-IB* |  |  |  | -0.26·  (0.13) |  |  |  |  |  |  |  |
| *First Donation* |  |  |  |  | 0.22·  (0.12) |  |  |  |  |  |  |
| *Block* |  |  |  |  |  |  | -0.25***  (0.07) |  |  |  |  |
| *Group Size* |  |  |  |  |  |  |  | 0.02  (0.21) |  |  |  |
| *Variance in Conformity* |  |  |  |  |  |  |  |  | -1.36*  (0.65) |  |  |
| *Computer Influence* |  |  |  |  |  |  |  |  |  | 1.45***  (0.13) |  |

*Note.* *** p<0.001; ** p<0.01; * p<0.05; · p<0.1; (SE). ^1^This model focuses on social influence trials only. ^2^This model caps influence magnitude at **Δ**.

*Table S17 Influence Magnitude (Age Continuous)*

|  | **RfM** | **RfM + Gender** | **RfM + Guess conversion rate** | **RfM + Abstract reasoning** | **RfM+ First donation** | **RfM + Converted Δ** | **RfM + Block order** | **RfM + Group size** | **RfM + Response variance** | **RfM^1^+ Non-social influence** | **RfM^2^** |
| --- | --- | --- | --- | --- | --- | --- | --- | --- | --- | --- | --- |
| *Intercept* | 2.02***  (0.25) | 1.9***  (0.28) | 2.02***  (0.24) | 2.01***  (0.25) | 2.12***  (0.25) | 2.32***  (0.21) | 2.52***  (0.29) | 2.02***  (0.25) | 2.45***  (0.34) | 2.05***  (0.24) | 2.03***  (0.2) |
| *Age Trend:* Linear | -0.95  (14.35) | -1.85  (19.14) | -0.06  (14.23) | -1.32  (14.27) | 4.27  (14.53) | -2.82  (17.53) | -1.3  (14.28) | -6.28  (18.52) | -5.73  (14.59) | -2.66  (15.95) | -4.17  (16.28) |
| *Age Trend:* Quadratic | 21.29  (14.37) | 22.77  (20.85) | 19.17  (14.28) | 15.06  (14.69) | 21.23  (14.3) | 32.59·  (17.55) | 21  (14.31) | 21.75  (14.41) | 23.2  (14.42) | 29.59·  (16.04) | 30.66·  (16.31) |
| *Direction:* Selfish | 0.73*  (0.33) | 0.74*  (0.33) | 0.73*  (0.33) | 0.75*  (0.33) | 0.5  (0.35) | 0.55  (0.34) | 0.73*  (0.33) | 0.74*  (0.33) | 0.75*  (0.33) | 0.5  (0.34) | 0.69*  (0.33) |
| *Source:* Adults | -0.26  (0.16) | -0.26  (0.16) | -0.26  (0.16) | -0.26  (0.16) | -0.26  (0.16) | -0.23·  (0.13) | -0.26  (0.16) | -0.26  (0.16) | -0.26  (0.16) | -0.26  (0.16) | -0.25·  (0.13) |
| *Source:* Computer | -0.9***  (0.19) | -0.9***  (0.19) | -0.9***  (0.19) | -0.9***  (0.19) | -0.9***  (0.19) | -0.88***  (0.13) | -0.91***  (0.19) | 0.90***  (0.19) | -0.90***  (0.19) |  | -0.9***  (0.13) |
| *Δ* | 0.85***  (0.14) | 0.85***  (0.14) | 0.85***  (0.14) | 0.85***  (0.14) | 0.9***  (0.14) | 1.6***  (0.23) | 0.85***  (0.14) | 0.86***  (0.14) | 0.86***  (0.14) | 1***  (0.16) | 0.86***  (0.14) |
| *Age Trend:* Linear x *Direction:* Selfish | 39.94  (28.99) | 40.35  (29.03) | 40.1  (28.98) | 40.79  (28.99) | 38.72  (28.94) | 27.59  (30.77) | 40.74  (28.79) | 40.04  (28.99) | 42.12·  (29.09) | 9.14  (30.28) | 35.02  (30.04) |
| *Age Trend:* Quadratic x *Direction:* Selfish | -9.85  (28.64) | -10.02  (28.67) | -9.34  (28.64) | -9.78  (28.64) | -8.79  (28.59) | -29.07  (30.22) | -7.99  (28.44) | -10.03  (28.64) | -10.58  (28.72) | -16.68  (29.86) | -16.99  (29.7) |
| *Age Trend:* Linear x *Δ* | -12.79  (9.92) | -12.71  (9.92) | -12.6  (9.93) | -12.58  (9.91) | -14.59  (9.94) | -11.55  (19.25) | -12.82  (9.94) | -12.91  (9.93) | -12.87  (9.92) | -24.54*  (11.72) | -12.67  (9.92) |
| *Age Trend:* Quadratic x *Δ* | 0.2  (9.95) | 0.21  (9.95) | -0.02  (9.95) | 0.37  (9.94) | 0.14  (9.93) | 10.9  (19.88) | 0.03  (9.97) | 0.3  (9.95) | -0.21  (9.94) | -6.07  (11.85) | 3.51  (9.94) |
| *Direction:* Selfish x *Δ* | 0.52***  (0.15) | 0.52***  (0.15) | 0.52***  (0.15) | 0.53***  (0.15) | 0.39*  (0.16) | 0.54*  (0.22) | 0.53***  (0.15) | 0.52***  (0.15) | 0.52***  (0.15) | 0.24  (0.18) | 0.48**  (0.15) |
| *Source:* Adults x *Δ* | 0.02  (0.13) | 0.02  (0.13) | 0.02  (0.13) | 0.02  (0.13) | 0.02  (0.13) | -0.04  (0.14) | 0.02  (0.13) | 0.02  (0.13) | 0.02  (0.13) | 0  (0.14) | 0.03  (0.13) |
| *Source:* Computer x *Δ* | -0.26*  (0.13) | -0.27*  (0.13) | -0.26*  (0.13) | -0.27*  (0.13) | -0.27*  (0.13) | -0.22·  (0.13) | -0.26·  (0.13) | -0.26*  (0.13) | -0.27*  (0.12) |  | -0.29*  (0.13) |
| *Age Trend:* Linear x *Direction:* Selfish x *Δ* | 42.7**  (14.44) | 42.75**  (14.46) | 42.69**  (14.43) | 42.8**  (14.43) | 42.02**  (14.44) | 56.04*  (23.61) | 42.69**  (14.44) | 42.83**  (14.45) | 42.84**  (14.45) | 39.04*  (17.92) | 40.93**  (14.62) |
| *Age Trend:* Quadratic x *Direction:* Selfish x *Δ* | 33.38*  (14.24) | 33.44*  (14.27) | 33.78*  (14.23) | 32.98*  (14.23) | 32.73*  (14.24) | 18.16  (22.05) | 33.69*  (14.24) | 33.13*  (14.25) | 33.69*  (14.26) | 35.66*  (17.7) | 31.83*  (14.41) |
| *Gender:* Male |  | 0.22  (0.26) |  |  |  |  |  |  |  |  |  |
| *Age Group:* Mid Adolescents x *Gender:* Male |  | 2.12  (23.13) |  |  |  |  |  |  |  |  |  |
| *Age Group:* Adults x *Gender:* Male |  | -3.22  (23.86) |  |  |  |  |  |  |  |  |  |
| *Conversion Rate Guess* |  |  | 0.31*  (0.13) |  |  |  |  |  |  |  |  |
| *Mars-IB* |  |  |  | -0.25·  (0.13) |  |  |  |  |  |  |  |
| *First Donation* |  |  |  |  | 0.23·  (0.12) |  |  |  |  |  |  |
| *Block* |  |  |  |  |  |  | -0.25***  (0.07) |  |  |  |  |
| *Group Size* |  |  |  |  |  |  |  | -0.08  (0.18) |  |  |  |
| *Variance in Conformity* |  |  |  |  |  |  |  |  | -1.24·  (0.65) |  |  |
| *Computer Influence* |  |  |  |  |  |  |  |  |  | 1.45***  (0.13) |  |

*Note.* *** p<0.001; ** p<0.01; * p<0.05; · p<0.1; (SE). ^1^This model focuses on social influence trials only. ^2^This model caps influence magnitude at **Δ**.

*Table S18. Adolescent participants only (Age Categorical)*

|  | First Donations | Influence Probability | Response Times | Influence Magnitude |
| --- | --- | --- | --- | --- |
| Intercept | 24.73***  (1.5) | -0.21  (0.17) | 8.09***  (0.05) | 2.50***  (0.36) |
| Age Group: Mid Adolescents | 1.54  (1.98) | -0.62**  (0.22) | 0.09  (0.06) | -0.73  (0.48) |
| Source: Adults |  | -0.07  (0.08) | 0.02  (0.02) | -0.24  (0.34) |
| Source: Computer |  | -0.54***  (0.08) | 0  (0.02) | -0.99**  (0.34) |
| Contagion: Not Influenced |  |  | -0.11**  (0.04) |  |
| Age Group: Mid Adolescents x Direction: Selfish |  | 0.01  (0.34) |  |  |
| Age Group: Mid Adolescents x Direction: Selfish x Contagion: Not Influenced |  |  | -0.12  (0.07) |  |
| Age Group: Mid Adolescents x Direction: Selfish x Δ |  |  |  | -0.78*  (0.33) |

*Note.* *** p<0.001; ** p<0.01; * p<0.05; · p<0.1; (SE). Estimates are Bonferroni corrected upon reviewer request.

*Table S19. Adolescent participants only (Age Continuous)*

|  | First Donations | Influence Probability | Response Times | Influence Magnitude |
| --- | --- | --- | --- | --- |
| Intercept | 67.47  (88.59) | -2.29***  (0.55) | 8.37***  (0.43) | 0.69  (2.7) |
| Age Trend: Linear | 7019.79  (14761.29) |  | 33.00  (64.02) | -209.37  (405.06) |
| Age Trend: Quadratic |  |  | 7.87  (28.48) | -53.09  (181.29) |
| Age Trend: Cubic | 1525.53  (3030.18) |  |  |  |
| Age Trend: Inverse |  | 25.28**  (7.82) |  |  |
| Source: Adults |  | -0.07  (0.08) | 0.02  (0.02) | -0.27  (0.16) |
| Source: Computer |  | -0.53***  (0.1) | 0  (0.02) | -1.03***  (0.16) |
| Contagion: Not Influenced |  |  | -0.7·  (0.35) |  |
| Age Trend: Inverse x Direction: Selfish |  | 8.21  (12.24) |  |  |
| Age Trend: Linear x Direction: Selfish x Contagion: Not Influenced |  |  | 93.86  (66.41) |  |
| Age Trend: Quadratic x Direction: Selfish x Contagion: Not Influenced |  |  | 48.61  (29.53) |  |
| Age Trend: Linear x Direction: Selfish x Δ |  |  |  | 831.56*  (323.51) |
| Age Trend: Quadratic x Direction: Selfish x Δ |  |  |  | 411.71**  (145.84) |

*Note.* *** p<0.001; ** p<0.01; * p<0.05; · p<0.1; (SE). Estimates are Bonferroni corrected upon reviewer request.
